# Combining modelling tools to evaluate a goose management scheme

**DOI:** 10.1007/s13280-017-0899-5

**Published:** 2017-02-18

**Authors:** Johannes M. Baveco, Anne-Kari Bergjord, Jarle W. Bjerke, Magda E. Chudzińska, Loïc Pellissier, Caroline E. Simonsen, Jesper Madsen, Ingunn M. Tombre, Bart A. Nolet

**Affiliations:** 1 Wageningen Environmental Research (Alterra) - Wageningen University and Research, PO Box 47, 6700 AA Wageningen, The Netherlands; 2 Division of Food Production and Society, Norwegian Institute of Bioeconomy Research (NIBIO), Box 115, 1431 Ås, Norway; 3 Norwegian Institute for Nature Research, FRAM – High North Research Centre for Climate and the Environment, PO Box 6606, Langnes, 9296 Tromsø, Norway; 4 Department of Bioscience, Aarhus University, Frederiksborgvej 399, 4000 Roskilde, Denmark; 5 Department of Environmental Systems Science, ETH Zurich, Zurich, Switzerland; 6 Department of Bioscience, Aarhus University, Kalø, Grenåvej 14, 8410 Rønde, Denmark; 7 Department of Animal Ecology, Netherlands Institute of Ecology (NIOO-KNAW), Droevendaalsesteeg 10, 6708 PB Wageningen, The Netherlands; 8 IBED, Theoretical and Computational Ecology, University of Amsterdam, Science Park 904, PO Box 94216, 1090 GE Amsterdam, The Netherlands

**Keywords:** Goose-agriculture conflict, Pink-footed goose, Refuge areas, Resource depletion model, Species distribution model, Yield loss

## Abstract

Many goose species feed on agricultural land, and with growing goose numbers, conflicts with agriculture are increasing. One possible solution is to designate refuge areas where farmers are paid to leave geese undisturbed. Here, we present a generic modelling tool that can be used to designate the best locations for refuges and to gauge the area needed to accommodate the geese. With a species distribution model, locations are ranked according to goose suitability. The size of the area to be designated as refuge can be chosen by including more or less suitable locations. A resource depletion model is then used to estimate whether enough resources are available within the designated refuge to accommodate all geese, taking into account the dynamics of food resources, including depletion by geese. We illustrate this with the management scheme for pink-footed goose *Anser brachyrhynchus* implemented in Norway. Here, all geese can be accommodated, but damage levels appear to depend on weather, land use and refuge size.

**Electronic supplementary material:**

The online version of this article (doi:10.1007/s13280-017-0899-5) contains supplementary material, which is available to authorized users.

## Introduction

As a result of shooting restrictions and improved foraging conditions on their wintering grounds and along their migratory route, many species of swans and geese (Anserinae) have increased during the last few decades in Europe (Fox et al. 2005, 2010) and North America (Ankney 1996; Abraham et al. 2005). As most of these species feed on agricultural land for a considerable part of their annual cycle, these population increases have led to more and more conflict with agriculture, and hence initiatives to control or compensate agricultural damage (Ankney 1996; Tombre et al. 2013a; Fox et al. 2016).

One mechanism to restrict damage and compensation payments is to create refuges where farmers are paid to accommodate geese to feed on their land undisturbed (Vickery and Gill 1999; Kwak et al. 2008). Two fundamental questions arise in relation to undertaking such measures: where these refuges should best be located and how large a refuge area should be allocated? Here, we propose to use a combination of species distribution modelling and resource depletion modelling, to tackle these questions. Species distribution models (SDMs) are tools to relate observations of species occurrence or abundance to environmental variables in order to predict a species distribution across a landscape (Elith and Leathwick 2009). Resource depletion models (RDMs) focus on the interplay between behavioural foraging decisions and the dynamic spatial distribution of resources (Gill et al. 2001). Used in combination, these models have the potential to guide the designation of refuges at the best locations and of the most appropriate size.

We here illustrate the combination of SDMs and RDMs with the management scheme developed to accommodate staging pink-footed geese *Anser brachyrhynchus* in central Norway. During spring, pink-footed geese congregate in Nord-Trøndelag county on migration to their breeding grounds on Svalbard. Here, the geese feed on farmland (Chudzińska et al. 2016b), causing a direct conflict of interests with the farmers. In response, farmers have been using different means of scaring the geese away. However, increasing complaints by farmers in the early 2000s led to the introduction of a subsidy system from 2006 onward by which farmers can be paid to accommodate the geese (Madsen et al. 2014; Norwegian Institute of Bioeconomy Research 2015) (see Fig. S1 for area involved).

In the management scheme, a SDM was used to rank areas according to their suitability for foraging pink-footed geese in Nord-Trøndelag (Jensen et al. 2008). The model was developed based on goose dropping presence/absence data from 2005 and predicted suitability as a function of environmental variables associated with disturbance, energy expenditure and start of the growing season. The model was successfully applied to prioritize fields (Madsen et al. 2014). For the present study, we further developed the SDM based on new data from 2011 enabling us to use dropping abundances rather than the presence/absence data per field.

One drawback of the SDM is that it does not account for within-season changes in resources at different sites, whereas this probably affects the habitat utilization of pink-footed geese staging in Nord-Trøndelag. During spring stopover, resources become available as the snow melts and grass starts to grow. Moreover, the action of farmers and the geese themselves affect resource availability between sites in the course of the growing season: farmers plough fields and sow new barley grains, the geese deplete left over grains on stubble fields and reduce sward heights on grass as the spring season progresses.

Such dynamics can be captured by RDMs. A multi-species, spatially explicit RDM was previously developed to determine whether sufficient refuge areas had been designated in the Netherlands to accommodate all overwintering geese and wigeon *Anas penelope* (Baveco et al. 2011). That model used goose and wigeon count data to set roost population sizes and simulated daily flights from roosts to the particular refuge site where they could balance their energy requirements at minimum energy cost. No resource growth occurred in winter, and resources were gradually depleted by the birds. Under such circumstances, the birds are predicted to first visit fields closest to the roost, albeit depending on the local resources, progressively using fields further away as those closest become depleted (Gill 1996). For the present study, we adapted the RDM to deal with pink-footed geese refuelling at the Nord-Trøndelag spring stopover site.

Combining the two modelling approaches, the ranking of suitability of fields for foraging geese produced by the SDM is used to delineate refuges, the total size of which depends on whether more or less suitable fields are included in the refuges. The RDM is then used to evaluate in detail whether sufficient resources are allocated within refuges during the whole stopover period. Under this approach, it is assumed that inside refuges pink-footed geese are allowed to forage unrestricted, while outside resources are not available, because the birds are scared away. The RDM simulates foraging and resource dynamics, incorporates agricultural management (ploughing, sowing) and accounts for the impact of weather conditions on all processes. Where grazing by geese can be translated into yield loss, there will be the possibility to estimate economic costs. The same is true for specific goose management measures (spring ploughing or delayed ploughing)—aimed at increasing resource availability to the geese—that may have an impact on barley yield. In the Nord-Trøndelag area, the lowered yield on grassland is a major factor in the conflict between geese and agriculture (Tombre et al. 2013b; Bjerke et al. 2014; Norwegian Institute of Bioeconomy Research 2015). We can obtain an estimate of yield loss from the RDM, by simulating the dynamics both with and without grazing and comparing the difference in standing crop at the end of the stopover period.

The aim of our study is to illustrate our approach and how it can be used to obtain an understanding of how the level of crop damage caused by geese depends on the interplay between weather conditions (snow melt and temperature), land use (crop type, timing of sowing and ploughing), the size of the designated refuge area and of the staging population. With the combined models, measures to increase the accommodation capacity of the refuge area and/or to decrease the level of expected crop damage may be evaluated.

## Materials and methods

### Study system

Pink-footed geese from the Svalbard population make a spring stopover (mid-April–mid-May) in the lowlands surrounding the interior part of the fiord Trondheimsfjorden in Nord-Trøndelag county in central Norway. The landscape is a mosaic of farmland and woodland. The farmland mainly consists of cultivated grassland dominated by timothy *Phleum pratense* L. and barley *Hordeum vulgare* L. cereal fields. Parts of the cereal fields are ploughed in autumn, but particularly on sloping terrain, stubbles with spilled barley grains remain until spring (Table 1). Stubble fields are ploughed on average around 3 weeks after the disappearance of the snow, when weather conditions have led to disappearance of ground frost and sufficiently dry soils. Roughly 1 week after ploughing, fields are newly sown with barley grains, providing low density of grains at the surface.

**Table 1:** Parameter values for modelling spring stopover of pink-footed geese in Nord-Trøndelag

Parameters	Units	Values	References
Body mass *M* (g)	g	2612	Baveco et al. (2011)
Grassland			Brands (2012)
Functional response *b*_*1*_	g m^−1^	0.28	(1)
Functional response *b*_*2*_	g m^−1^	9.6	
Functional response *c*	s m^−1^	2.8	
Minimal cropping time *T*_c0_	s	0.42	
Maximal chewing rate *R*_max_	g s^−1^	0.0085	
Alert factor		1.00	
Cereal fields			B.A. Nolet (unpubl. data)
Attack rate *a*	m^2^ s^−1^	0.00325	
Handling time *h*	s g^−1^	69	
Metabolism			
Basal metabolic rate BMR	J s^−1^	8.69	Baveco et al. (2011) (2)
Resting metabolic rate RMR (=1.4 BMR)	J s^−1^	12.2	Baveco et al. (2011)
Field metabolic rate FMR (=1.9 BMR)	J s^−1^	16.5	Baveco et al. (2011)
Flight metabolic rate VMR (=14.2 BMR)	J s^−1^	123.4	Madsen and Klaassen (2006) (3)
Energy intake for weight increase *E*_G_	J day^−1^	1 235 621	(4)
Flight speed *v*	m s^−1^	13.9	Chudzińska et al. (2016b)
Max. distance from roost *V*_max_	m	10 000	C.E. Simonsen (unpubl. data)
Max. goose density	ind ha^−1^	350	C.E. Simonsen (unpubl. data)
Twilight used for foraging	h	1	B.A. Nolet (unpubl. data)
Foraging periods	Day^−1^	2	Chudzińska et al. (2016b)
Resource data			
Initial values (April 1)			
Grass LAI	m^2^ leaves m^−2^ soil	0.6	
Grass biomass leaves, stems	g DW m^−2^	42.9	
Barley grains (stubble fields)	g DW m^−2^	21.6	(5)
Field management			
Fraction spring ploughing	–	0.5	Chudzińska et al. (2015)
Ploughing delay	Day	22 (SD 7)	Supplementary Material 1.5
Sowing delay	Day	10 (SD 4)	Supplementary Material 1.5
Sowing density (barley)	m^−2^ (g DW m^−2^)	450 (23.9)	(6)
Available fraction sown	–	0.13	(7)

(1) Based on biomass per plant, bite size on *Phleum* taken to be 2.8× greater than on *Lolium* (http://www.bioforsk.no/ikbViewer/Content/35578/Liv.pdf)

(2) Mistakingly given as 7.35

(3) 8.9 J m^−1^ × 13.9 m s^−1^

(4) *c* = *e*_g_· Δ*m*· *LBM*/*k*_g_, where energy tissue content *e*_*g*_ = 27 500 J g^−1^ (Madsen and Klaassen 2006), fraction body mass increase Δ*m* = 0.0157 (Lindström 1991), lean body mass LBM = 2382 g (Madsen and Klaassen 2006) and efficiency of utilization of metabolizable energy *k*_*g*_ = 0.83 (Lopez and Leeson 2005)

(5) 408 grains m^−2^ × 0.053 g DW grain^−1^ (own measurements)

(6) Recommended sowing density barley 450 grains m^−2^. Dry weight grain 53 mg (own measurements)

(7) To arrive at the 60 grains m^−2^ density measured at soil surface (Chudzińska, personal communication)

Numbers of pink-footed geese in Nord-Trøndelag peak in the first ten days of May, when complete roost counts carried out in 2010, 2012 and 2013 totalled 60 646, 65 024 and 73 905, respectively. During these years, around 45 main roosts were used by the birds (Table S1). Individual geese stay about 3 weeks in the area, first foraging on stubble fields, gradually moving to grassland and newly sown cereal fields; ploughed fields do not provide any resources (Chudzińska et al. 2016b).

### Species distribution model

#### Field protocol

We randomly selected 10 points located within areas known to be used by pink-footed geese in Nord-Trøndelag and considered all agricultural fields within a radius of 1 km of each selected point, resulting in a sample size of 290 fields within the 10 blocks. During two subsequent days at the end of the spring staging period, we visited all 290 fields, after which we excluded fields not subject to goose damage (stubble and ploughed fields, and the cereal fields where barley had not started sprouting yet). This left 175 grasslands and 64 cereal fields where goose damage had potentially occurred. In each field, droppings were counted in three 2-m-radius circles placed in the field at the centre, 2/3 and 1/3 from the nearest source of disturbance, e.g. the road. Furthermore, we measured grass sward height with a 25-cm plastic disc (weight 59 g) sliding down a measuring stick (Stewart et al. 2001; Simonsen et al. 2016). We undertook three random height measurements within each of the three circles, totalling nine registrations per field.

#### Goose foraging habitat selection

Goose dropping abundance represents a proxy for the time spent by geese in a given field and thereby grazing pressure on each field (Owen 1971; Patterson et al. 1989). We modelled the relationship between dropping density and a set of environmental variables including geophysical, environmental, climatic (Pellissier et al. 2013) and anthropogenic features, factors that are relevant for the field preferences by geese (Table S10).

#### Statistics

We ran a linear mixed model with a Quasi-Poisson distribution using the glmmPQL call from the MASS package (Venables and Ripley 2002) in R version 2.14.0 (R Development Core Team 2013) to account for the random effect of the 10 blocks in the sampling design. In addition, we ran a general linear model not including the block effect to compare whether the estimated parameters differed with and without considering the block effect. We initially investigated 17 variables for respective correlation and considered only one variable within pairs where correlation values were >0.8. We ran a step-wise model selection procedure based on Akaike Information Criterion (AIC).

We validated the predictive ability of the models using a split-sampling approach partitioning the data into 70% for model training and 30% testing data, while ensuring that each block contributed equally to each set of data. Our observational data were all within c. 4 km of roosting sites and we chose not to predict beyond 8 km from roost, because all fields are within that range of a roost (Chudzińska et al. 2016a). On some of the smaller islands in the fjord, there are no registered roads and buildings, and as the distance to disturbance was an important explanatory variable in the model, we also excluded these areas.

### Resource depletion model

The RDM simulated the months April and May, and required as input a definition of the initial available resources (on grassland and cereal fields) contained in refuges, the size of the pink-footed geese population at each day and the daily weather conditions. Refuge size could range from all suitable fields in Nord-Trøndelag to small subsets of the most suitable fields, depending on the chosen level of suitability (the predicted dropping densities from the SDM), see Scenarios section below.

#### Resources

Resources became available after snow melt (Tables S8, S9). Fields in the refuges were either grassland or cereal fields, with cereal fields being either unploughed stubble fields or autumn-ploughed fields that did not provide any resources to the geese until all cereal fields had been ploughed and were newly sown. Grass growth on cultivated grasslands was modelled using a simplified version of the CATIMO model (Bonesmo and Bélanger 2002) assuming optimal (non-limiting) water and nitrogen conditions (Supplementary Material Section 1.3).

Resource distribution was obtained from spatial datasets including land use map AR5 (Norwegian Institute of Bioeconomy Research 2015), dropping counts (Simonsen 2014) and field surveys (Chudzińska et al. 2015). Distinguishing between grassland and cereal fields was only possible for roughly half of the fields. For the other fields, use was set in a probabilistic way, with the probability of a field being a cereal field obtained from the annual agricultural statistics at municipality level (Statistics Norway 2015) (Table S2). Spring ploughing of cereal fields was set with a fixed probability (0.5) (Statistics Norway, data from Nord-Trøndelag county in 2010).

#### Bird data

The seasonal pattern in the pink-footed geese numbers during their stay in Nord-Trøndelag was derived from roost counts made from 2005 to 2007. We scaled the numbers to the estimated annual maximum number of birds present, and used a 4th-order polynomial fit through the average seasonal pattern to define the relative abundance at day *x* (Fig. S2):

1 $$ y = a_{4} \cdot x^{4} + a_{3} \cdot x^{3} + a_{2} \cdot x^{2} + a_{1} \cdot x + a_{0} $$

With $a_{4} = 4.6985\, \times \,10^{ - 7}$, $a_{3} = - 8.2776\, \times \,10^{ - 5}$, $a_{2} = 2.4601\, \times \,10^{ - 3}$, $a_{1} = 2.3456\, \times \,10^{ - 2}$ and $a_{0} = - 4.8315\, \times \,10^{ - 2}$ (day number *x* = 1 at April 1).

The total number of birds in the area at any day in any year was set by multiplying relative abundance at that day (Eq. 1) with the annual maximum number (see “Study system” section above; for 2009 we used 60 646 and for 2011 62 835). 45 main roosts used in the periods 2005–2007 and 2009–2013 were identified (Table S1; Fig. S1). The daily distribution of birds over roosts was assumed to be proportional to the amount of resources accessible from each roost.

#### Weather data

Weather data were obtained for nine weather stations in the region (Norwegian Meteorological Institute 2015) (Table S5). Data included average wind speed (m s^−1^) and mean, minimum and maximum daily temperatures (°C), from which daily radiation (10^7^ J m^−2^ day^−1^) was derived (Tables S6, S7; Figs. S5, S6). For each field, weather data of the nearest weather station were used.

Snow cover data were obtained from three of the weather stations. The average first day without snow was used (Table S9). With every 25 m elevation (above 50 m), an additional day was added to this date. Elevation values at the centre of each field were obtained from digital elevation model data at 50 m resolution (Norwegian Mapping Authority 2014). The times between the first day without snow and the days farmers could start to plough or to sow were derived from data provided by a number of individual farmers in the area (Table S3; Fig. S4).

#### Foraging model

For the current spring model, birds would choose the field where an aspirational weight gain (as opposed to energy balance as in the winter model; Baveco et al. 2011) could be realized against the lowest costs; this seems reasonable as weight increase over the stopover period was constant and not maximal (Chudzińska et al. 2016b). This implies that, on a daily basis, metabolizable energy intake (MEI, J day^−1^) should equal energy expenditure (DEE, J day^−1^) increased by the scope for weight gain (*E*_G_, J day^−1^). The required foraging time $T_{f}^{*}$ [Eq. 3 in (Baveco et al. 2011)] was therefore changed to:

2 $$ T_{\text{f}}^{*} = \frac{{\left( {T - T_{V} } \right){\text{RMR}} + T_{V} {\text{VMR}} + E_{G} }}{{qe{\text{IIR}} - ({\text{FMR}} - {\text{RMR}})}}, $$

where *T* is the fixed total day length (s), *T*_*V*_ the time (s) spent flying from and to a roost, RMR is the resting metabolic rate (J s^−1^), VMR is the metabolic rate in flight (J s^−1^), FMR is the field metabolic rate (J s^−1^) depending on weather, IIR is the instantaneous intake rate (g s^−1^), *q* assimilation efficiency and *e* the energy content (J g^−1^) of the food. As tracks of individuals with satellite transmitters indicated that they usually had two separate foraging periods each day, with a visit to the roost at mid-day, the distance-dependent flight time and thus flight costs, were doubled (Chudzińska et al. 2016b). Apart from these two aspects, the model was used as described in (Baveco et al. 2011) and shortly summarized in the following.

For all fields within a 10-km radius of a roost, $T_{\text{f}}^{*}$ was calculated. The fields for which the required foraging time was shorter than the day length (daylight period) were considered as potential foraging sites, and from this set, the one with the smallest DEE was selected as the ‘optimal’ foraging field. DEE amounted to

3 $$ {\text{DEE}} = T_{\text{f}}^{*} \left( {{\text{FMR}} - {\text{RMR}}} \right) + \left( {T - T_{V} } \right){\text{RMR}} + T_{V} {\text{VMR}}. $$

The model requires temperature, radiation and wind speed at each field to calculate the field metabolic rates.

Intake rates depend on resource type and resource levels. For grass a type 4 functional response was used, with intake rate depending on sward height *L* (m):

4 $$ {\text{IIR}}_{(L)} = \frac{1}{\alpha }\left[ {\frac{{1 + b_{2} L}}{{b_{1} L}}\left( {T_{c0} + cL} \right) + \frac{1}{{R_{ \hbox{max} } }}} \right]^{ - 1} . $$

In Eq. 4, *α* represents a correction factor for alert time (s), *b*_*1*_ and *b*_*2*_ are regression coefficients determining bite size depending on *L*. *T*_c0_ and *c* determine cropping time depending on *L*, while *R*_max_ represents the maximum rate of chewing (g s^−1^). For barley grains collected from the soil surface, a type 2 functional response was assumed, with attack rate *a* (m^2^ s^−1^) and handling time *h* (s g^−1^), related to grain density *D* (g m^−2^) according to

5 $$ {\text{IIR}}_{(D)} = \frac{aD}{1 + ahD}. $$

Parameter values are given in Table 1.

#### Grazing impact

In order to convert total above-ground grass biomass into sward height and vice versa, the relationship height (m) = biomass (g m^−2^)/1640 was used (Mould 1992). The calculated amount grazed by the geese (in g m^−2^) was divided by the total biomass (leaves plus stems) present, and this fraction was used to proportionally decrease leaves biomass, stems biomass and leaf area index (LAI; the three state variables of the grass growth model).

The economic impact of grazing was quantified as yield loss at the time of the first harvest. This was obtained from the model by simulating in the same run, for each grassland field, grass growth with and without grazing by geese. Without grazing simulates an ‘exclosure’ where grass growth is driven by weather conditions only. At any time, yield loss can be calculated as the difference between the two grass biomass (g m^−2^) variables and converted to total biomass by multiplying by field size.

#### Testing

For model testing, we compared calculated goose days per area, cumulated over the whole stopover period, to dropping counts performed in 2011 (Simonsen 2014). We also compared the predicted distribution of geese over roosts to those from the counts in 2010, 2012 and 2013 (Table S4). Both comparisons were done for a range of refuge sizes.

### Scenarios

The suitability values from the SDM were used to prioritize fields for inclusion in the set of refuges, and hence the total area assigned as refuge depended on the suitability value being used as cut-off value. For a range of cut-off values, the RDM was applied to quantify whether sufficient resources were available within these refuges over the whole staging period, focusing on accommodated goose days, resource use and yield loss. The situation with all fields included in the refuges (thus cut-off set to zero) served as a reference, indicating the potential accommodation capacity of the area as a whole. Simulations were run for each of the five (2009–2013) sets of weather and snow conditions, using the population size estimated for each of these years.

For two hypothetical maximum population sizes (60 000 and 140 000), we ran simulations with each of the five (2009–2013) sets of weather and snow conditions, to account for annual variability in these conditions. The two sizes were chosen, because 60 000 is the population target defined in the International Species Management Plan (ISMP) for the Svalbard population of pink-footed geese and 140 000 is the projected population size in 2022 (the year of revision of the ISMP) if no management actions were taken to control the population size (J. Madsen and F.A. Johnson, personal communication). We assumed that the entire population is concentrated in Nord-Trøndelag during a few weeks in spring.

## Results

### Species distribution model

The most parsimonious species distribution model contained the following explanatory variables: perimeter/area ratio of each field, distance to roosting sites, distance to disturbance (roads and buildings), precipitation in April, sward height, available habitat in a 1000-m radius and a categorical variable indicating whether the field was intensively grown crop or non-cultivated pasture. Slope, solar radiation and minimum temperature in April did not contribute to the model exploratory power, and were not included in the final model (Table S10). While contributing to explain dropping counts, we excluded sward height in the final model because this variable was not known over the entire area and could therefore not be used for projections. Both models, GLM (cor = 0.5087) and GlmmPQL (cor = 0.5087) showed good predictive abilities to predict independent data during the split-sample validation procedure. Both the linear and the mixed model showed similar estimated parameters, had similar predictive abilities and were largely comparable (Table 2, Tables S11 and S12), and we used the linear model for the spatial projections.

**Table 2:** Results from the GLM model of dropping density. Predictor variables are precipitation in April (prcp4), perimeter/area ratio (periarea), distance to roads and buildings (non-agri), distance to roost (roost), minimum temperature in April (tmin4), percentage of available habitat in 1000 m radius (nb1000) and the authority label on field (as.factor(agri))

	Estimate	SE	*t* value	Pr(>|*t*|)
(Intercept)	−3.077e+00	2.927e+00	−1.051	0.294
prcp4	6.812e−02	3.955e−02	1.722	0.086
periarea	−2.771e+01	8.866e+00	−3.125	0.002**
non-agri	3.354e−03	1.121e−03	2.992	0.003**
roost	−6.694e−04	1.634e−04	−4.096	5.96e−05***
tmin4	9.476e−02	8.091e−02	1.171	0.243
nb1000	2.748e−02	1.038e−02	2.648	0.009**
as.factor(agri)	1.446e+00	1.009e+00	1.434	0.153

### Resource depletion model

**Fig. 1:**
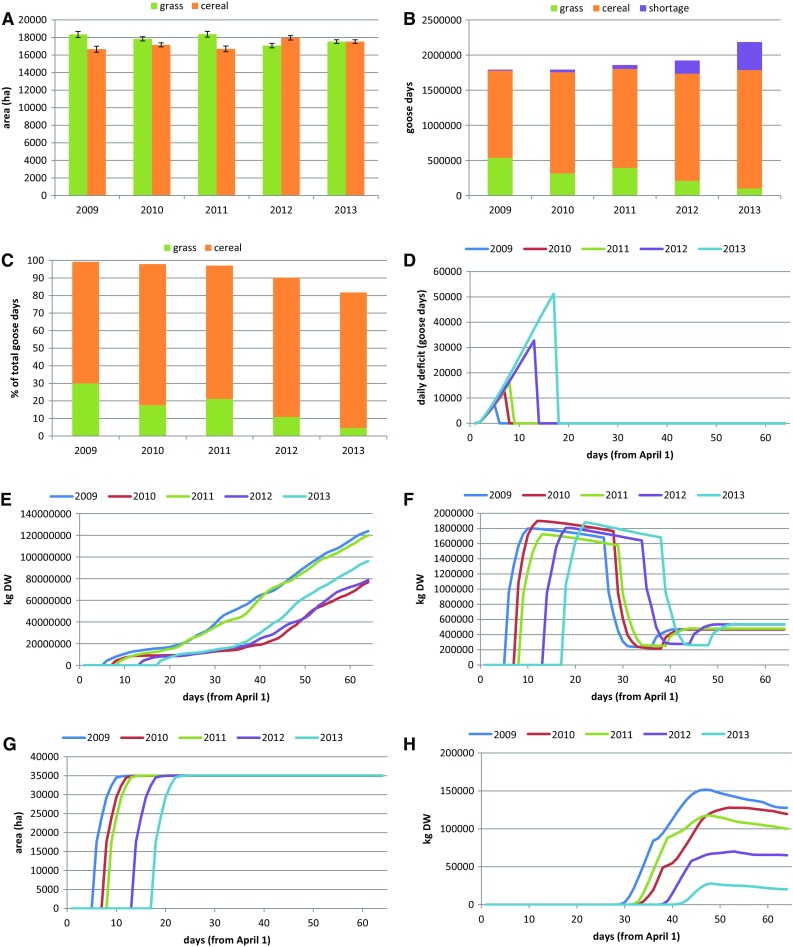
**a** Areas of grassland and barley cereal fields available to goose grazing in spring in Nord-Trøndelag, mid Norway. **b** Goose days accommodated on grass and stubble fields and the shortages (not accommodated goose days). **c** Goose days accommodated as percentages of total number of goose days spent in the area. **d** Unaccommodated goose days. **e** Available grass (kg DW). **f** Available grains (kg DW on stubble fields and newly sown fields). **g** Area of cereal fields and grassland without snow cover (ha). **h** Estimates of grass yield loss (kg DW). All values are averages over 5 runs of the resource depletion model applied for 2009–2013 for the reference case (all fields available)

For the reference situation, with all resources accessible, in total, c. 17 500-ha grassland and about the same area of cereal fields were available in Nord-Trøndelag (Fig. 1a). Whether the complete stopover population of pink-footed geese could be accommodated depended on the year: the accommodated fraction was highest in the early spring of 2009 and lowest in the late spring of 2013 (99 and 81%, respectively; Figs. 1b, c). Deficits (i.e. goose days that could not be accommodated) occurred only at the beginning of the staging period (Fig. 1d) and built up as long as most fields were still covered by snow (Fig. 1g). After complete snow thaw, grass growth was still slow due to generally cool weather, causing this resource to increase slowly (Fig. 1e), while barely grains on stubble fields were immediately available (Fig. 1f). The total amount of predicted yield loss on grassland (Fig. 1h) was lowest in the late spring of 2013, when the geese did not use grasslands as much as in the earlier years, because stubble fields were longer available. In all years, yield loss decreased again towards the end of the staging period, due to the decelerating (saturating) nature of grass growth that set in earlier in the non-grazed than in the grazed situation.

The model predicted that barley grains on stubble fields constituted the main resource (Fig. S7). When stubble fields were ploughed and/or to a certain degree depleted, the birds switched to grass, except when this was not yet available (such as in the late spring of 2013). A second peak in grain consumption was predicted, resulting from the appearance of newly sown fields. Apparently, model settings were such that the birds preferred newly sown fields over grassland, when both were available. The impact of the underlying assumptions on seed densities was checked in a sensitivity analysis (Figs. S15–S20).

The relationship between predicted density of (cumulative) goose days (m^−2^) to dropping counts indicates that the RDM was not very precise in defining the exact fields where foraging would be intense (2011; Fig. S11A). The relatively low relationship between predicted and observed distributions over roosts (2010, 2012, 2013; Figs. S11B–D) suggested that the model spread the geese between more roosts than observations imply.

### Combination of Species Distribution Model and Resource Depletion Model

**Fig. 2:**
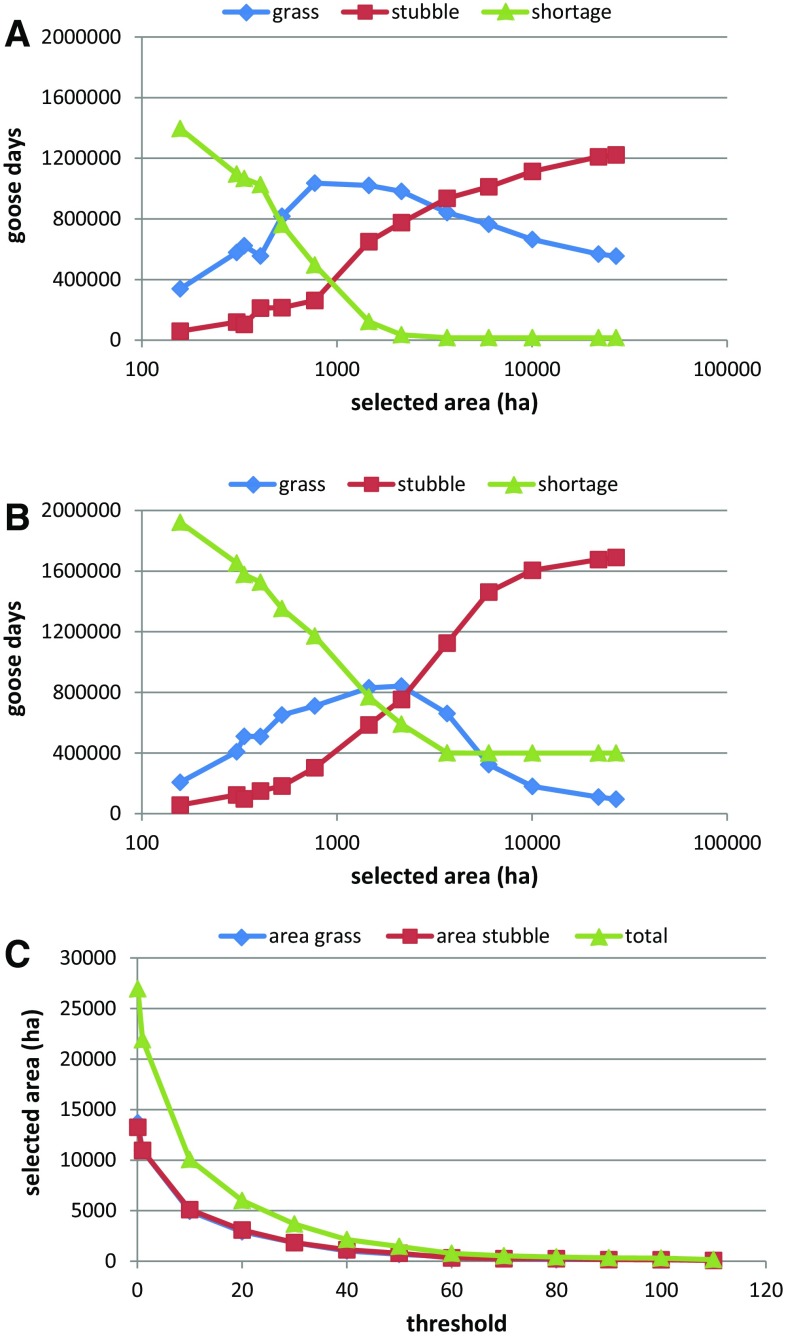
Results of running the resource depletion model on an increasing refuge area, when fields are added following the prioritization suggested by the species distribution model (SDM). Total numbers of goose days accommodated on grass and cereal fields, and the shortage (unaccommodated goose days) for 2009 (**a**) and 2013 (**b**). **c** The relationship between selected refuge area distinguishing between grassland, cereal fields and total area, and the applied threshold value for suitability. All values are averages over 5 runs

**Fig. 3:**
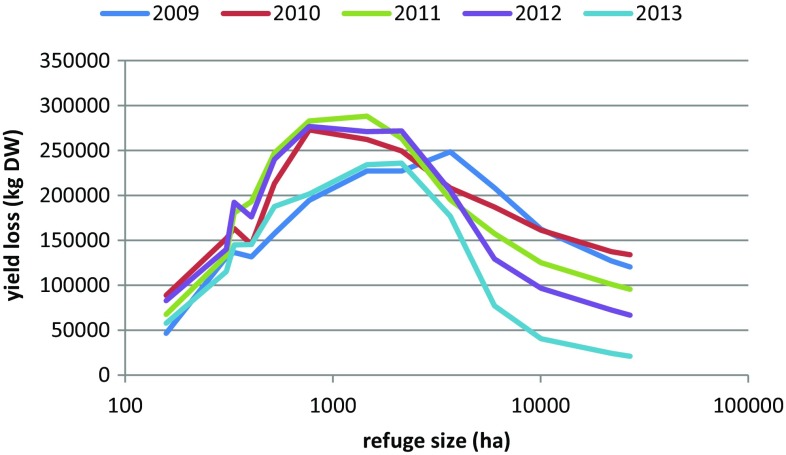
Total yield loss of grass biomass (kg DW) at the end of the simulated period (day 64), depending on refuge size, for each of the years (averaged over 5 runs)

As expected, the capacity of the Nord-Trøndelag area to accommodate the birds increased with the size of the refuges (Fig. 2 and Fig. S8). With the threshold set to 40–50 droppings, 2000–4000 ha of approximately equal fractions of grassland and cereal fields (Fig. 2c) became available that could accommodate most goose days. However, late resource availability due to prolonged snow cover (as in 2012 and 2013) cannot be compensated for by adding more refuge area. Although 2000–4000 ha may suffice to avoid deficits, with this refuge size, the exploitation of grassland was high, leading to a relatively high yield loss (Fig. 3). With increasing size of the refuges and hence more cereal fields being accessible, the birds exchanged grass for barley grain consumption. When all fields were accessible (the reference case), almost the entire accommodated population (2013) relied on barley grain (Figs. 1b, c, 2).

The fit between model results and dropping counts (2011) and counts at the roosts (2010, 2012 and 2013) did not improve at all when only highly prioritized fields were included in the refuges (Figs. S12 and S13).

### Projections

**Fig. 4:**
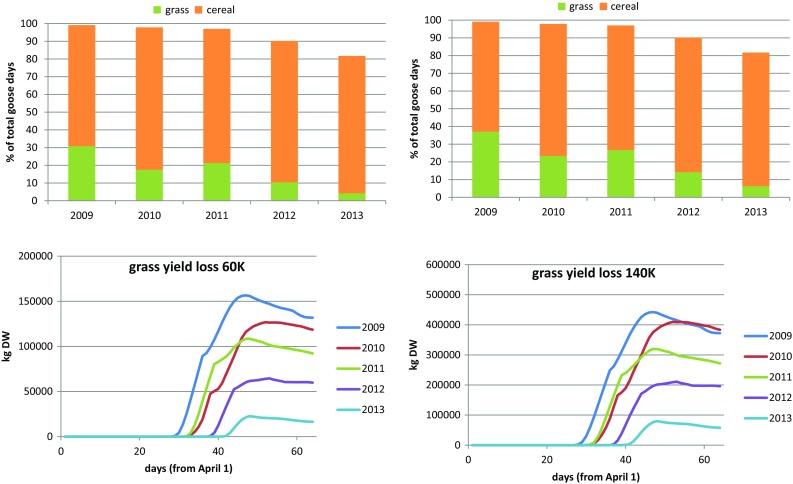
(*top*) Percentages of accommodated goose days on barley cereal fields and grassland, for maximum population size 60 000 (*left*) and 140 000 (*right*), combined with each weather dataset. (*bottom*) Grass yield loss dynamics for 60 000 (*left*) and 140 000 (*right*) population size, for all weather sets. Reference case with all fields available

For the reference case (all fields available), for both population sizes, the number of accommodated birds depended mainly on weather conditions (Fig. 4). When the population was larger (140 000), barley grain consumption reached a maximum slightly earlier, causing grain depletion and the switch to grass to take place earlier as well (Fig. S9). Predicted yield losses for the large population were therefore relatively higher, more than proportional to bird numbers (Fig. 4 bottom). Apart from the difference in timing, for large and small population sizes the consumption patterns were similar (Fig. S9).

**Fig. 5:**
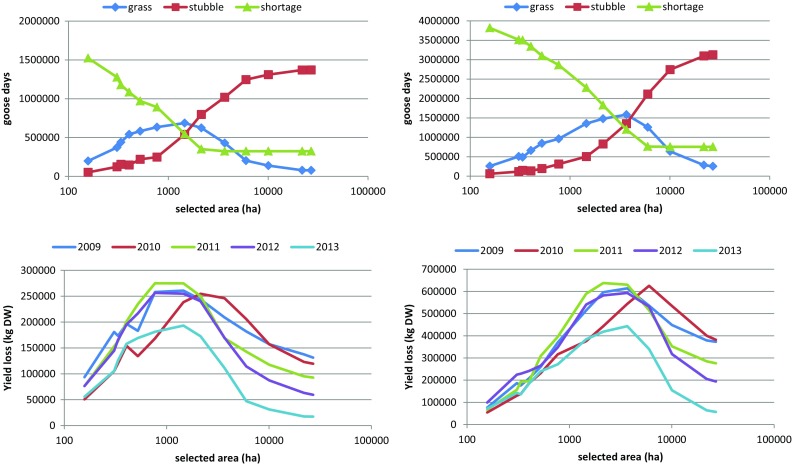
(*top*) Numbers of goose days accommodated on grassland and barley cereal fields, and shortages, using the 2013 weather dataset (see Fig. 2). (*bottom*) Yield loss at the end of the staging period (day 64), depending on refuge area, for all the 5 weather datasets

With smaller refuge sizes (higher threshold values) (Fig. 5) the same pattern emerged for the two hypothetical population sizes as for estimated population sizes (Fig. 2). With lowered threshold values and thus an increase in refuge area, more geese could be accommodated and as soon as not all of both available resources was needed anymore, grass was exchanged for barley grain. Yield loss therefore reached a maximum at intermediate refuge size (Fig. 5 bottom). The shape of the curves differed for the two population sizes, with the larger population (140 000) reaching maximum yield loss at considerably larger refuge size. Until the refuge size at which deficits levelled off (Fig. 5 top), resources were apparently limiting; above this size, the geese could actually choose between grass or barley grain consumption. Increasing refuge area from 770 to 3672 ha (Fig. S10) allowed the small population to increase their grain consumption relatively much more than the larger population, which still needed all available resources.

## Discussion

Two major insights were obtained from the application of our combined approach. Firstly, barley grains on stubble fields appeared to play a crucial role in supporting foraging geese, being the only resource available at the beginning of the stopover period. Information on whether a field represented grassland, or a spring- or autumn-ploughed cereal field becomes crucial, at least when predictions on a field-by-field basis are made. Secondly, weather conditions determined to a large extent whether all geese could be accommodated and what yield loss would result from goose grazing. The timing of the disappearance of the snow cover had the largest impact, while after snow melt weather conditions determined the grass growth rate and thus when the grass could substitute barley grain in the birds’ diet.

Comparison of dropping counts and field-specific goose days predicted by the RDM indicated that the model was not very good at predicting foraging intensity on a field-by-field basis (Fig. S11). This was however expected, as more than half of all the fields in the region were randomly set to cereal field or grassland (Fig. S14), while the model appeared very sensitive to the area of cereal (stubble) fields in the early part of the staging period. Although these were not the fields associated with the dropping counts (because for these fields, the actual crop status was known), the status of other fields in the neighbourhood of a particular field is bound to have a large impact on its use. To derive the best predictions from the model, we conclude that precise information on the status of each field is indispensable, although we doubt whether the model will be able to predict goose usage at such a fine scale. The geese are modelled to have perfect knowledge, whereas other attempts to model goose spacing have found that a better fit with the data was obtained when imperfect knowledge was assumed (Amano et al. 2006; Chudzińska et al. 2016a).

The distribution between roosts should be less dependent on the precise state of each field. The model predicted this distribution reasonably well. However, in all cases, the model birds were distributed over many roosts, while in the counts, it was evident that they were more concentrated on a few roosts. In the model, the distribution was made proportional to total resource availability around each roost. Thus, it is likely that this assumption does not hold, and birds concentrate more than proportional to available resources and/or other factors besides resource quantity determine their distribution as well, such as the benefit of flocking (Amano et al. 2006) or effects of disturbance causing aggregation of flocks.

With regard to the role of annually variable and unpredictable weather conditions, the applied methods need to take these into account by adding safety margins around all model-based predictions. These might be obtained by applying multiple weather and snow condition datasets (“weather scenarios”). For simplicity, we assumed that the phenology of the geese did not depend on whether a year had an early or late spring, but some flexibility with regard to spring conditions is seen in the departures from Denmark and Nord-Trøndelag (Tombre et al. 2008; Duriez et al. 2009).

### Combination of species distribution model and resource depletion model

We believe that the proposed combination of modelling tools to locate the most suitable refuge sites of sufficient size to accommodate the geese would be a major step forward in reducing conflict with farmers. Application of the SDM will help to locate the refuge sites at locations were the geese actually go, whereas application of the RDM may help to avoid refuges that are too small to support the geese. Both aspects are important considering that some previous goose accommodation schemes did not work. In the Netherlands, geese were not concentrating in the refuge areas despite scaring campaigns (Schekkerman et al. 2012), and in Scotland, geese were spilling over from refuge areas because they were (becoming) too small (Cope et al. 2003). Scaring campaigns have to be rather intense before they will be effective in keeping geese away from farmland outside refuges (Percival et al. 1997; Tombre et al. 2005; Simonsen et al. 2016).

### Projections

The model showed that the numbers of geese that could be accommodated in the region, and the damage these geese could cause to agriculture, depended on the interplay of three main factors: weather conditions, agricultural management including land use and the size of the refuges. Firstly, the weather (including snow cover and temperature) conditions determined when the first resources became available to the geese and at what rate the grass began to grow and hence when it was able to provide a food alternative for the geese. Secondly, the area of stubble fields that were not ploughed in the preceding autumn was critical in determining the amount of resources in the early goose staging period, when grass growth had not yet started. When stubble fields are depleted by the geese or ploughed by the farmers, grasslands become proportionally important for the geese. Thirdly, the size of the refuges was important in that the set of refuges had to include both enough stubble fields (for resources becoming available early in the stopover season) as well as grassland (for resources being available later in the staging period). Together the three factors determined the resulting patterns in resource consumption by the geese (Fig. S7). Patterns could vary from one with predominantly barley grain consumption to one with up to three alternating peaks in grain and grass consumption (Figs. S7 and S19).

Potential grass yield loss in the model was directly linked to grass consumption. As the model birds could obtain more energy from stubble fields, they switched only to grassland when barley stubble fields were depleted or ploughed. With a lower density of grains in stubble fields, the switch would have occurred earlier, and the damage would have been larger. Spring-ploughing probability determining stubble field area and barley grain density on stubble fields were thus crucial model coefficients. Their impact on the results was explored in a sensitivity analysis that suggested that over a wide range of realistic values more or less the same results would be expected. Final yield loss was modelled as the difference in grass biomass between model runs with and without geese at a given date, so under the assumption that harvest date was the same. This is the same approach as used in an experimental study of yield loss by grazing pink-footed geese in Nord-Trøndelag (Bjerke et al. 2014). In reality, farmers might want to postpone the first harvest when the grasslands have been heavily grazed, in which case the yield loss may be not so much in terms of biomass, but more so in terms of possibilities to do a second and third harvest.

The insights obtained from application of the models suggest possible modifications to the management scheme, aimed at increasing the accommodation capacity while lowering grass yield loss. Firstly, all stubble fields in the area should be made available to the geese, instead of including only a subset of them in designated refuges. This seems to be already done in practice, as in the last years subsidies were granted only for grasslands. Secondly, to increase the area of stubble fields, autumn ploughing could be discouraged in favour of spring ploughing. Thirdly, ploughing in spring could be postponed so that the geese can maximize their use of stubble fields. This will however only be effective when stubble fields are not already depleted at the earliest sowing dates, implying that stubble fields should be abundant. Delayed sowing of barley has not been found to lead to cereal yield loss later (Riley et al. 2005). If cereal yield loss would occur, it should be taken into account in the modelling, to balance the costs of grass yield loss, cereal yield loss and of goose management and to arrive at truly optimal solutions.

## Conclusion

Increasing goose populations cause more and more conflicts with agriculture all across the northern hemisphere. One way to limit these conflicts is to designate accommodation areas for geese. We advocate a combination of modelling tools to determine the optimal location and size of refuges to accommodate geese. We illustrate the approach for the spring stopover of pink-footed geese in Nord-Trøndelag (Norway), where the geese feed mainly on cereal fields and grasslands. Apart from refuge size, spring weather conditions and land use practices appear to have the largest effects on number of accommodated geese and subsequent grass yield loss. Focussing on management issues, the main option in this specific area seems to be adjusting the ploughing practice. By restricting the ploughing of stubble fields in autumn, barley leftovers become available to the geese in the following spring. A similar effect is achieved by postponing ploughing in spring. Specific subsidies would be required to stimulate such a practice, as the farmers currently do not want to forego any possibility to plough the stubble fields as soon as possible. Our combined modelling tools may be a useful in many other situations where geese and farmers meet.

## Electronic supplementary material

Below is the link to the electronic supplementary material.

Supplementary material 1 (PDF 3084 kb)
