# Supplementary material for: Combining modelling tools to evaluate a goose management scheme
Source: Ambio. 2017 Feb 18;46(Suppl 2):210–23. doi: 10.1007/s13280-017-0899-5 (PMC5316330; doi:10.1007/s13280-017-0899-5)
Supplement: Supplementary file 1 — Supplementary material 1 (PDF 3084 kb) [file 13280_2017_899_MOESM1_ESM.pdf]

Ambio

Electronic Supplementary Material

This supplementary material has not been peer-reviewed

Title: **Combining modelling tools to evaluate a goose management scheme**

Authors: Hans (J.) M. Baveco, Anne-Kari Bergjord, Jarle W. Bjerke, Magda E. Chudzińska, Loïc Pellisier, Caroline E. Simonsen, Jesper Madsen, Ingunn M. Tombre, Bart A. Nolet

CONTENTS

1 SUPPORTING INFORMATION FOR RDM APPLIED TO NORD-TRØNDELAG 2
1.1 Roosting Sites 2
1.2 Goose Phenology 5
1.3 Grass Growth 6
1.4 Land Use 8
1.5 Ploughing and sowing delays 11
1.6 Roost Counts 13
1.7 Weather 15
1.8 Snow cover 18
1.9 References 19
2 RESULTS FOR RDM & SDM APPLIED TO NORD-TRØNDELAG 20
2.1 Resource Consumption & Accommodated Numbers 20
2.2 Comparison with Counts 24
2.3 Species Distribution Model 28
3 SENSITIVITY ANALYSIS RDM APPLIED TO NORD-TRØNDELAG 30

1 SUPPORTING INFORMATION FOR RDM APPLIED TO NORD-TRØNDELAG

1.1 Roosting Sites

The model required a (point) shape file with roosting sites as input. Roost locations (Table S1) were defined in geographic coordinate system GCS-WGS-1984, and converted to UTM. For roost locations on the map, see figure S1.

Table S1 The 45 main roosting sites in Nord-Trøndelag in the periods 2005-2007 and 2009-2013.

Roost name	Roost ID	longitude	latitude	UTM-X	UTM_Y
Alstadhaugbukta, Alfnesbukta	5	11.212618	63.721409	609283.65	7067862.98
Bartnes	34	11.214262	64.048303	608100.75	7104275.25
Bosnes	32	11.322384	63.891625	614013.46	7087013.02
Eidsbotn	6	11.247096	63.728487	610958.29	7068710.74
Eidsvatnet	24	12.171231	64.554674	651980.69	7162624.66
Ekne	8	11.044948	63.697514	601089.67	7064925.49
Fiborgtangen, Hotterbukta	41	11.147736	63.709393	606125.00	7066415.22
Fossemvatnet	19	11.638833	64.062538	628754.39	7106649.95
Frøset	17	11.410927	63.973089	618015.65	7096247.02
Gjørv	15	11.343427	63.937454	614858.58	7092154.97
Klingsundet, Kvam, Stod	21	11.739777	64.137416	633318.45	7115195.97
Korsen	14	11.373186	63.948677	616270.11	7093458.85
Kroksvågen	16	11.374242	63.968962	616237.67	7095719.99
Kvamsholman	43	11.211704	63.855228	608722.09	7082766.07
Leksdalsvatnet north	31	11.576332	63.917830	626357.79	7090409.95
Leksdalsvatnet south	9	11.634364	63.832284	629596.32	7080999.93
Lorvikleiret	10	11.374919	63.883203	616626.61	7086170.04
Lundavatnet	25	11.455570	64.085339	619718.16	7108831.73
Lundleira, Egge	18	11.437357	64.023847	619092.67	7101949.17
Lømsen, Følling	37	11.518574	64.097793	622733.75	7110338.66
Lønnem	13	11.382472	63.946555	616733.74	7093239.49
Mære church	33	11.376202	63.937676	616463.50	7092239.14
Naust	12	11.372589	63.926647	616332.20	7091004.21
Nordskaget	45	11.418955	63.823854	619039.01	7079641.52
Overhalla,	40	11.837886	64.446504	636552.85	7149822.69

Homstad					
Ranemsletta	44	11.948378	64.490131	641640.84	7154922.73
Reinsvatnet	36	11.565715	64.034465	625314.26	7103377.86
Rinnleiret	29	11.425935	63.773631	619594.97	7074061.33
Risan	1	11.244269	63.868209	610271.54	7084267.72
Røra	3	11.397031	63.847028	617863.50	7082181.76
Semsøra, Snåsa	20	11.579068	64.105544	625645.52	7111319.84
Snåsa	39	12.359939	64.254364	662788.19	7129660.13
Stjørdal, Vikanbukta	38	10.781417	63.477367	588748.14	7040012.45
Sundneshamn	42	11.271445	63.868329	611605.92	7084328.37
Tynestangen north	28	11.339120	63.767089	615343.29	7073173.04
Tynestangen south	7	11.326931	63.758206	614778.51	7072161.76
Vellamelen	22	11.379247	64.110709	615892.86	7111516.07
Vellamelen, Hjellösen	23	11.315475	64.115997	612766.17	7111990.52
Vellamelen, Strandabukta	35	11.351548	64.110628	614544.45	7111456.92
Verdalselva north	4	11.469849	63.799862	621646.01	7077065.57
Verdalselva south	30	11.482174	63.783176	622325.08	7075230.86
Vika, Sund	2	11.313654	63.854259	613735.99	7082835.80
Våsetbukta	11	11.371367	63.916186	616315.64	7089836.91
Ørin north	26	11.435766	63.811427	619918.80	7078289.05
Ørin south	27	11.454856	63.801560	620900.57	7077226.19

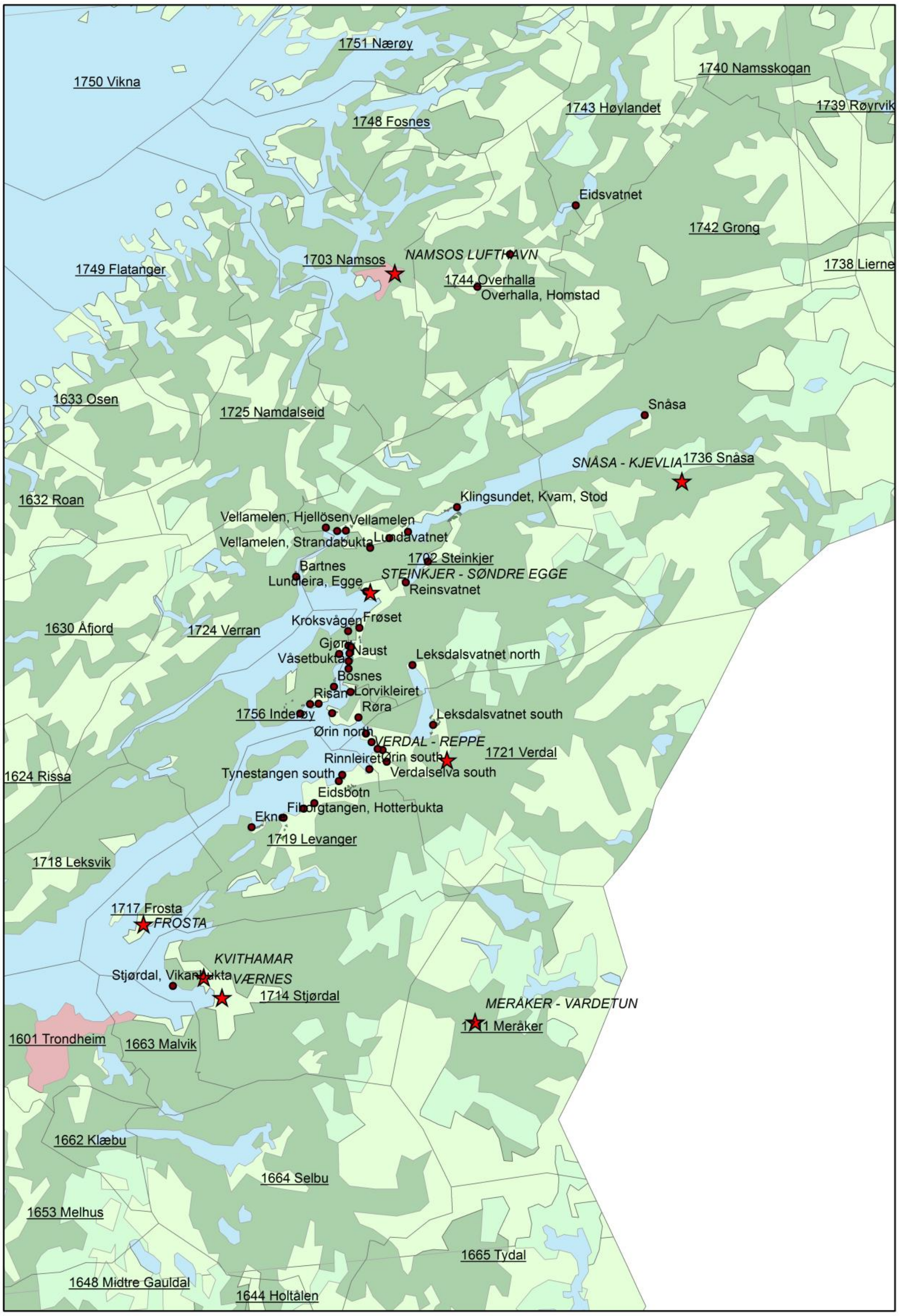

Fig. S1 Roosts (red circles), weather stations (red stars) and municipalities (identified by their underlined names) in the staging area. Green shades obtained from the Norwegian topographic map (http://www.norgeskart.no/#6/340184/7073037).

1.2 Goose Phenology

The phenology (the general pattern in the abundance of the pink-footed geese during the six weeks of their stay in Trøndelag) was derived from counts in 2005 to 2007. Counts were performed from cars or elevated points in the terrain, by experienced observers by use of telescopes and binoculars. By scaling the numbers on the estimated maximum number of birds present in/around the middle of the six weeks period, comparable patterns were obtained for each year (Fig. S2). The 4th-order polynomial that was fit through the average pattern gives a reasonable approximation of the temporal pattern in relative abundance of the pink-footed geese. The maximum number of birds present in the area was set to the maximum number of birds observed at the annual count.

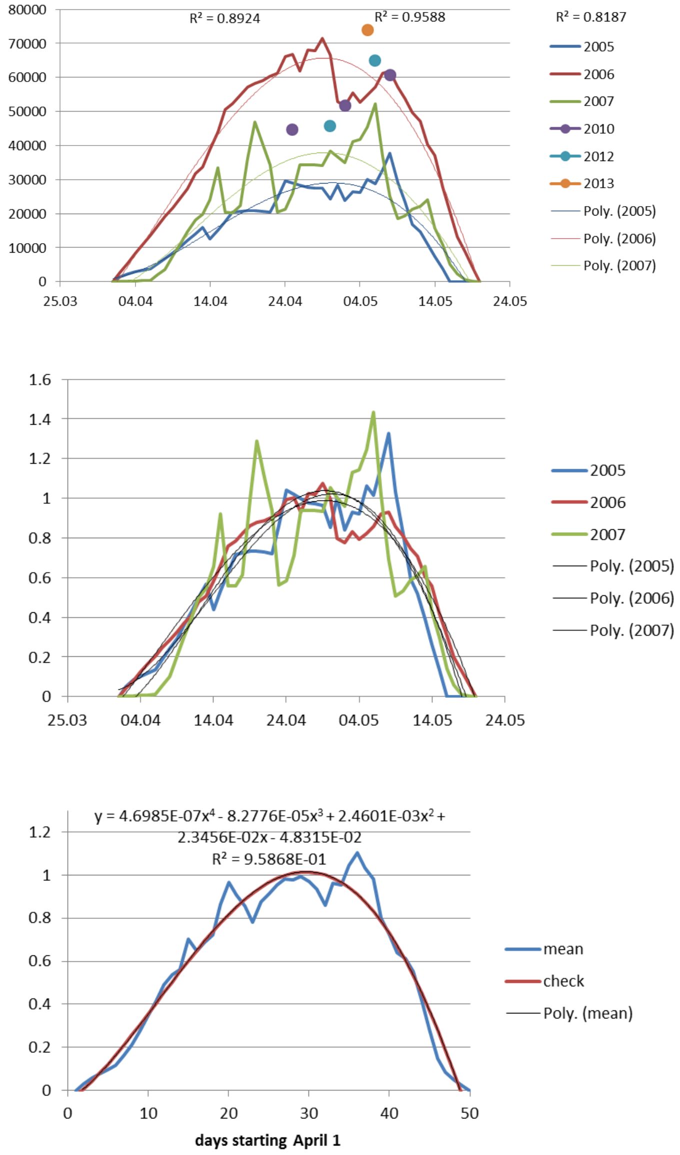

Fig. S2 Top: the total numbers during the period April 1 to May 20, for 2005, 2006 and 2007, and the single counts for the years 2010, 2012 and 2013. R^2 values for 2005, 2006 and 2007 (left to right). Middle: numbers scaled on the estimated population size in the middle of the period, for each year. Bottom: average of the scaled numbers with fitted 4th degree polynomial.

1.3 Grass Growth

For grass growth on cultivated grasslands we implemented the CATIMO model (Bonesmo and Bélanger 2002) for timothy (*Phleum pratense* L.), assuming optimal (non-limiting) water and nitrogen conditions. With daily values for temperature (mean daily value) and PAR (photosynthetic active radiation), see section 1.7 Weather, this model allowed us to predict the development in LAI (leaf area index, m^2 leaves m^{-2} soil), and biomass of leaves and stems (g m^{-1} DM).

As initial values at April 1, we assumed a LAI of 0.6, and leaves and stems biomass 46.7 and 2.5 g m^{-2} DM, respectively (A.-K. Bergjord, *personal communication*). To test the model, we applied it on the experiment described in (Bjerke et al. 2013), using temperature and radiation data of the nearest (<30 km) weather station (Mære, lat: 63.9425, long:11.4255), from:

http://lmt.bioforsk.no/agrometbase/getweatherdata.php?showValueTypeSelect=true

The radiation data on the site were provided in MJ m^{-2} d^{-1}. The result is shown in Figure S3. The model initially underestimated biomass. This might be due to initial values being set unrealistically low (zero). For the later dates in 2011, the model overestimated biomass, which might be due to the assumptions of optimal water and nitrogen conditions. For the other years, the fit appeared reasonable.

The onset of growth is assumed to be at the first passage of the 5 day diurnal mean air temperature of 5°C (Bonesmo 1999). Bergjord suggested that the condition might be a bit stricter, requiring the 5 day mean value to be over 5°C for three consecutive days (*personal communication*).

No data are available on the impact of grazing on LAI and biomass of leaves and stems. In the model, the functional response was based on grass length. The relationship length (m) = biomass (g m^{-2} DM) / 1640 was used to convert biomass into grass length and vice-versa (Mould 1992). Note that we did not use the exact same equation, as the original one predicted zero biomass at a grass-length of 2 cm (yield would be zero at this length). The calculated amount grazed by the geese (in g m^{-2} DM) was divided by the total biomass (leaves plus stems) present, and this fraction was then used to proportionally decrease leaves biomass, stems biomass and LAI.

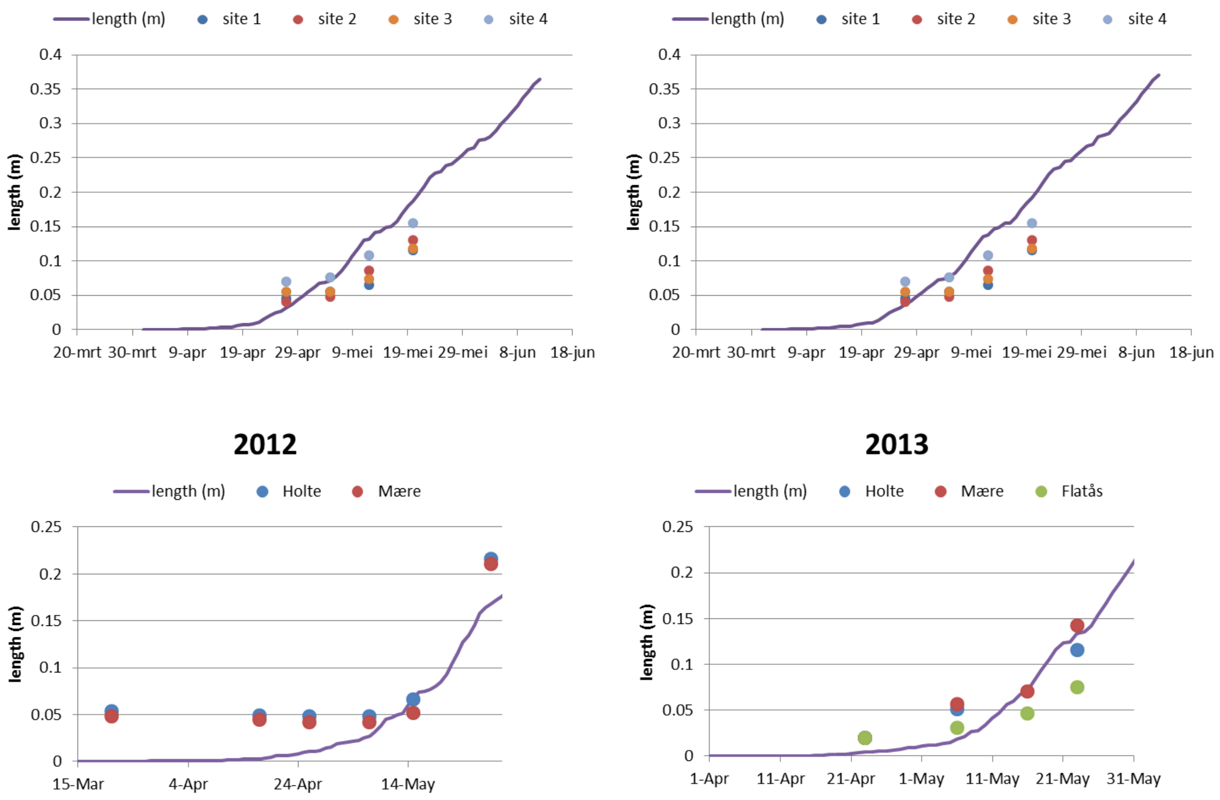

Fig. S3 Top row: the predicted grass length compared to the grass length estimated at 4 experimental sites in 2011 (Bjerke et al. 2013). Top-left: growth started at April 7th, when 5 day mean temperature was above 5ºC for 3 consecutive days. Top-right: growth started at April 5th, when 5 day mean temperature exceeded 5ºC for the first time. Bottom row: values on two or three locations for 2012 and 2013, both with the condition 5 day mean temperature exceeded 5ºC for the first time.

1.4 Land Use

From the basic land use map AR5 (Norwegian Institute of Bioeconomy Research 2015), fields with relatively intensive agricultural use were selected, leading to the same set of resource fields used with both the species distribution model and the resource depletion model. The information on whether, in a specific year, a field was a cereal field or grassland was incomplete. The state of fields was known, in order of relevance, for those for which a subsidy was applied for in a given year (2009−2013), where dropping counts were performed in 2011 (Simonsen 2014) or which were included in a field survey in 2012 (Chudzińska et al. 2015) in total roughly half of the area). For the remaining unspecified fields, use was set in a probabilistic way, with the probability of a field being a cereal field obtained from the annual agricultural statistics at municipality level (Statistics Norway 2015) (Table S2). For cereal fields, spring ploughing was set with a fixed probability (0.5) (Statistics Norway, data from Nord-Trøndelag county in 2010).

We downloaded the agricultural statistics for N and S Trøndelag from:

https://www.ssb.no/statistikkbanken/selectvarval/Define.asp?subjectcode=&ProductId=&MainTable=JordbrukAreaA&nvl=&PLanguage=1&nyTmpVar=true&CMSSubjectArea=jord-skog-jakt-og-fiskeri&KortNavnWeb=stjord&StatVariant=&checked=true

We assumed that ”Cultivated land” and “Grain and oil seeds” approximated to the total (pastures + cereal) and the cereal field areas. In the model, when no information on a field’s agricultural use was available, the ratio of the areas “Grain and oil seeds” / “Cultivated land” was used to define the probability that the field was used to grow cereals. Few oil seed rape fields were present in the area, potentially biasing the ratio (we checked this for some of the largest agricultural municipalities).

Table S2 Agricultural area in decares (0.1 ha), by municipality and year. For each municipality the first row refers to total cultivated land (emboldened text), and the second to grain and oil seeds (italics). Missing data are indicated by dots. For these municipalities we assumed no cereal fields to be present

municipality	**2009**	**2010**	**2011**	**2012**	**2013**
	**Agricultural area**	**Agricultural area**	**Agricultural area**	**Agricultural area**	**Agricultural area**
**1601 Trondheim**	**55198**	**55178**	**54370**	**53853**	**53908**
	39892	*39933*	*39450*	*38858*	*38727*
**1612 Hemne**	**18214**	**17664**	**17959**	**18152**	**18102**
	..	..	..	..	..
**1613 Snillfjord**	**11037**	**10898**	**10876**	**10987**	**10814**
	0	*0*	*0*	*0*	..
**1617 Hitra**	11161	11014	9973	9456	9338
	..	..	..	..	..
**1620 Frøya**	**4095**	**4537**	**4776**	**4696**	**3352**

	0	*0*	*0*	*0*	*0*
**1621 Ørland**	**38457**<br>*20841*	**38515**<br>*21364*	**38477**<br>*22157*	**38574**<br>*22148*	**38229**<br>*21652*
**1622 Agdenes**	**15993**<br>*1566*	**15682**<br>*1624*	**15854**<br>*1597*	**15865**<br>*1391*	**15598**<br>*1221*
**1624 Rissa**	**53277**<br>*13719*	**52538**<br>*13872*	**49568**<br>*13872*	**49844**<br>*13662*	**50075**<br>*13551*
**1627 Bjugn**	**30581**<br>*5461*	**28867**<br>*5378*	**28902**<br>*5647*	**28691**<br>*5542*	**28350**<br>*5299*
**1630 Åfjord**	**27539**<br>*2561*	**27373**<br>*2726*	**25917**<br>*2774*	**26216**<br>*2488*	**25668**<br>*2351*
**1632 Roan**	**9461**<br>*688*	**9502**<br>*655*	**9683**<br>*625*	**9724**<br>*647*	**9304**<br>*537*
**1633 Osen**	**7029**<br>*..*	**6973**<br>*..*	**6852**<br>*..*	**6527**<br>*0*	**6918**<br>*0*
**1634 Oppdal**	**40215**<br>*493*	**40412**<br>*611*	**40539**<br>*583*	**40438**<br>*..*	**40683**<br>*962*
**1635 Rennebu**	**27891**<br>*1544*	**27316**<br>*1785*	**26588**<br>*1669*	**26376**<br>*1595*	**26380**<br>*1488*
**1636 Meldal**	**27675**<br>*5684*	**27761**<br>*6099*	**27790**<br>*5989*	**28172**<br>*5977*	**28328**<br>*5350*
**1638 Orkdal**	**34516**<br>*9146*	**34386**<br>*9670*	**34924**<br>*9748*	**34256**<br>*9202*	**34251**<br>*8475*
**1640 Røros**	**21076**<br>*0*	**21286**<br>*0*	**20943**<br>*0*	**21310**<br>*0*	**21183**<br>*0*
**1644 Holtålen**	**14428**<br>*0*	**14268**<br>*0*	**14267**<br>*0*	**14177**<br>*0*	**14324**<br>*0*
**1648 Midtre Gauldal**	**46735**<br>*1033*	**46950**<br>*1061*	**46420**<br>*1075*	**46303**<br>*1107*	**46563**<br>*1035*
**1653 Melhus**	**65907**<br>*34789*	**66087**<br>*35351*	**65344**<br>*34780*	**64945**<br>*34098*	**64709**<br>*33569*
**1657 Skaun**	**29373**<br>*14464*	**26598**<br>*14072*	**26699**<br>*14107*	**26913**<br>*14293*	**27060**<br>*13770*
**1662 Klæbu**	**8661**<br>*3938*	**8092**<br>*3825*	**8057**<br>*4046*	**8074**<br>*3958*	**7579**<br>*3621*
**1663 Malvik**	**12837**<br>*6298*	**12696**<br>*6497*	**12122**<br>*6293*	**11990**<br>*6224*	**11725**<br>*5847*
**1664 Selbu**	**30965**<br>*6986*	**30711**<br>*6734*	**30971**<br>*6739*	**30981**<br>*6820*	**30316**<br>*6780*
**1665 Tydal**	**7470**<br>*0*	**7713**<br>*0*	**7444**<br>*0*	**7452**<br>*0*	**7845**<br>*0*
**1702 Steinkjer**	**152089**<br>*75871*	**150257**<br>*77670*	**150767**<br>*77328*	**151534**<br>*78095*	**152786**<br>*77788*
**1703 Namsos**	**17758**<br>*2250*	**17150**<br>*2454*	**16603**<br>*2779*	**15663**<br>*2579*	**15679**<br>*2315*

**1711 Meråker**	**7988**<br>*1197*	**8011**<br>*1091*	**8038**<br>*942*	**7873**<br>*869*	**7933**<br>*771*
**1714 Stjørdal**	**80196**<br>45866	**80845**<br>44997	**80563**<br>44803	**80678**<br>44498	**80041**<br>44745
**1717 Frosta**	**22248**<br>*10710*	**21951**<br>*10495*	**21768**<br>*10310*	**21928**<br>*9933*	**22459**<br>*9995*
**1718 Leksvik**	**20069**<br>*871*	**19986**<br>*915*	**17139**<br>*833*	**17331**<br>*853*	**17480**<br>*874*
**1719 Levanger**	**127919**<br>*64739*	**122094**<br>*64239*	**121922**<br>*63205*	**123116**<br>*63646*	**123205**<br>*63148*
**1721 Verdal**	**77108**<br>*39243*	**77708**<br>*39306*	**77717**<br>*39025*	**77289**<br>*38883*	**76717**<br>*37935*
**1724 Verran**	**8989**<br>*397*	**9256**<br>*465*	**9023**<br>*494*	**9149**<br>*479*	**9145**<br>*539*
**1725 Namdalseid**	**28314**<br>*4543*	**28358**<br>*5688*	**29344**<br>*5517*	**29456**<br>*7152*	**29416**<br>*6126*
**1736 Snåase Snåsa**	**33013**<br>*9277*	**33101**<br>*9405*	**33203**<br>*9924*	**33539**<br>*10394*	**33471**<br>*10199*
**1738 Lierne**	**14209**<br>*0*	**14827**<br>*0*	**14700**<br>*0*	**12933**<br>*0*	**12826**<br>*0*
**1739 Raarvihke Røyrvik**	**3163**<br>*0*	**3092**<br>*0*	**3085**<br>*0*	**2938**<br>*0*	**2985**<br>*0*
**1740 Namsskogan**	**6771**<br>*0*	**6724**<br>*0*	**7248**<br>*0*	**6775**<br>*0*	**6662**<br>*0*
**1742 Grong**	**18203**<br>*5451*	**17681**<br>*4915*	**17660**<br>*4902*	**17520**<br>*4607*	**17528**<br>*4315*
**1743 Høylandet**	**16568**<br>*2180*	**17033**<br>*2066*	**17122**<br>*2626*	**17114**<br>*2659*	**17155**<br>*2335*
**1744 Overhalla**	**42578**<br>*15771*	**40821**<br>*15018*	**41013**<br>*15365*	**41569**<br>*15238*	**41721**<br>*15303*
**1748 Fosnes**	**8856**<br>*654*	**8949**<br>*510*	**8206**<br>*601*	**7961**<br>*463*	**7880**<br>*468*
**1749 Flatanger**	**9260**<br>*808*	**9307**<br>*909*	**7906**<br>*792*	**8049**<br>*887*	**7945**<br>*855*
**1750 Vikna**	**14078**<br>*..*	**13790**<br>*..*	**13369**<br>*..*	**14366**<br>*..*	**12525**<br>*..*
**1751 Nærøy**	**37431**<br>*2220*	**36635**<br>*2089*	**34675**<br>*2339*	**34084**<br>*2428*	**33574**<br>*1639*
**1755 Leka**	**7115**<br>*71*	**6998**<br>*..*	**7119**<br>*..*	**7173**<br>*..*	**7351**<br>*..*
**1756 Inderøy**	**0**<br>*0*	**0**<br>*0*	**0**<br>*0*	**52267**<br>*27624*	**52342**<br>*27208*

.

1.5 Ploughing and sowing delays

Data on ploughing and sowing dates were obtained from 5 individual farmers in the area (Figure S4 and Table S3). In cases where the farmer provided a range of dates, the first date was used. For ploughing in days after snow disappearance the outlier year 2014 was ignored. In 2014 snow cover disappeared already beginning of March. For sowing, one outlier datum was ignored (sowing at day 168, 44 days after ploughing).

The fraction of cereal fields having been ploughed in the preceding autumn, and thus present as ploughed fields at the start of the staging period, was default set to 0.5, based on Chudzińska et al. (in press), estimated for a subset of the fields in the area. Data from Nord-Trøndelag county in 2010 show the same value (Statistics Norway).

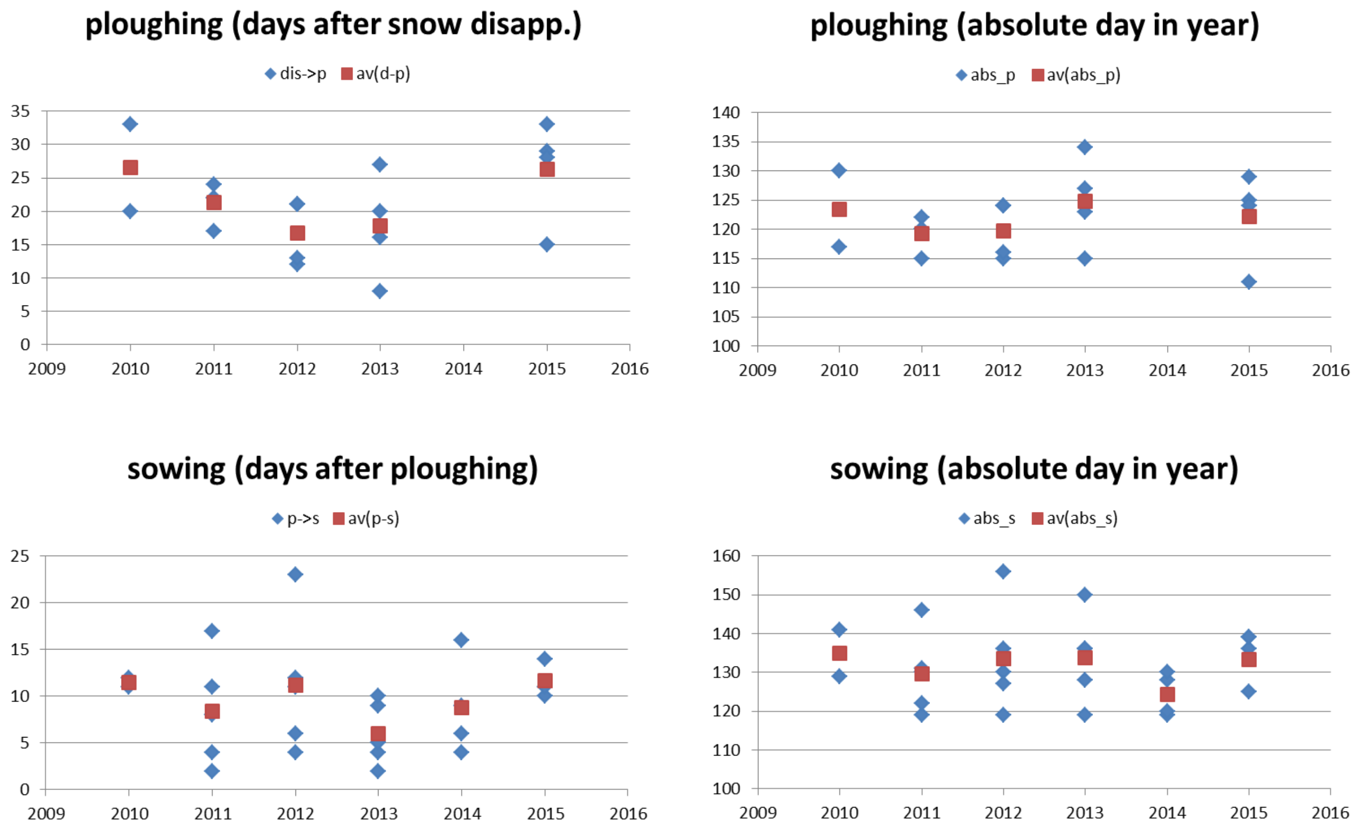

Fig. S4 Ploughing and sowing data for the individual farmers and averaged per year. Top row: ploughing date as days after snow disappearance (left) and day number (right). Bottom row: sowing date as days after ploughing (left) and day number (right).

	av(d-p)	av(p-s)	av(abs_p)	av(abs_s)
2010	26.5	11.5	123.5	135
2011	21.3	8.4	119.3	129.6
2012	16.8	11.2	119.8	133.6
2013	17.8	6	124.8	133.8
2014		8.8		124.3
2015	26.3	11.7	122.3	133.3
Average	21.7	9.6	121.9	131.6

Table S3 Average delays and dates for ploughing and sowing. Columns left to right: average delays in ploughing (days after snow disappearance); average delay sowing (days after ploughing); average day number for ploughing; average day number for sowing.

1.6 Roost Counts

Counts of goose numbers using known roost sites in Nord-Trøndelag were performed by a group of experienced observers on specific days during the period of peak occurrence of geese in 2010, 2012 and 2013. Counts were made in the middle of the day when most birds are known to concentrate on roost sites.

Table S4 Counts at the roosting sites in 2010, 2012 and 2013.

year	2010	2010	2010	2012	2012	2013
date	25-4	2-5	8-5	30-4	6-5	5-5
daynr	25	32	38	30	36	35
RoostID						
1	1600	375	2400	730	1041	1980
2	931	1400	28	566	475	68
3	0	0	0	1110	4200	550
4	3500	4333	2741	1371	14500	4400
5	30	63	279	0	0	1760
6	0	300	582	0	0	8000
7	2500	500	0	0	0	0
8	1320	1159	1839	0	0	3100
9	6278	6500	2500	900	4100	650
10	230	3400	1600	2050	0	2000
11	660	0	3000	893	1860	1800
12	0	45	1810	4000	0	0
13	0	0	440	0	0	2700
14	0	93	800	220	209	30
15	6	0	1406	639	1450	340
16	0	310	99	0	460	263
17	4500	83	1255	0	0	2200
18	3000	5130	1154	3280	1200	920
19	0	0	18	0	0	4000
20	1500	3500	2600	174	300	1315
21	5472	1185	374	6500	8000	671
22	2500	6300	1650	2700	7000	9000
23	1827	0	2250	578	0	0
24	0	0	300	0	0	1000
25	0	0	0	0	0	0
26	500	250	0	1362	1400	0
27	1000	3345	852	6100	0	870
28	1012	1000	905	895	4000	1000
29	1750	3258	3000	1500	4500	6800
30	0	2396	0	0	0	0
31	0	0	4000	1000	0	0
32	0	0	510	0	0	0
33	0	0	0	0	0	0
34	650	1804	450	1650	750	1150

35	0	0	3000	4200	3500	2000
36	0	0	0	0	0	0
37	0	0	0	640	0	0
38	0	0	0	600	750	0
39	0	0	0	0	0	0
40	0	0	0	0	0	1600
41	1487	1331	3435	0	0	8700
42	0	1200	0	133	0	0
43	0	0	0	0	630	0
44	0	0	0	0	0	2000
45	0	0	500	0	0	0

1.7 Weather

Norwegian weather data were obtained from eKlima (www.eklima.no) for 9 weather stations in the area (Table S5). The weather data that were available included average wind speed (m/s) and mean, minimum and maximum daily temperature (°C). Hours of sunshine were not recorded at most stations.

Daily radiation was thus calculated separately, using Angot's values (the solar radiation that would be received in case of a transparent atmosphere) assuming Northern Latitude of 60 °(Table 14 in van Keulen & Wolf 1986, Fig. S5) and using Hargreaves equation (Table S6). NB these values are in 10^7 J m-2 d-1.

Day length is calculated separately, using the value for days after winter solstice and latitude (Table S7).

As a check, we compared observed global radiation at Bioforsk Mære station 2012 against model-calculated values, applying temperature data from a nearby weather station (Steinkjer 71000) (Fig. S6).

Table S5 The nine weather stations that provided weather data. For 2010, Frosta data were not available

Stnr	Name	Altitude	Latitude	Longitude	Municipality
69100	VÆRNES	12	63.4592	10.9352	STJØRDAL
69150	KVITHAMAR	40	63.4882	10.8795	STJØRDAL
69380	MERÅKER – VARDETUN	169	63.4115	11.7277	MERÅKER
69655	FROSTA	70	63.5657	10.6940	FROSTA
70150	VERDAL – REPPE	81	63.7823	11.6742	VERDAL
70850	SNÅSA – KJEVLIA	195	64.1587	12.4692	SNÅSA
71000	STEINKJER - SØNDRE EGGE	6	64.0225	11.4508	STEINKJER
71780	ÅFJORD II	20	63.9662	10.2158	ÅFJORD
72580	NAMSOS LUFTHAVN	2	64.4708	11.5705	NAMSOS

.

Table S6 Smalltalk code for calculating the incoming daily global solar radiation (RAD) and Photosynthetically Active Radiation (PAR) using Hargreaves equation with Angot's values.

```
setRadHargreavesEquationForDayBeginningInApril: anInteger year: year
	"set Incoming daily global solar radiation, (Rg) here called rad [MJ m-2 d-1] and Photosynthetic
	Active Radiation, par [MJ m-2 d-1]"
	"use Angots from the formula in van Keulen & Wolf 1986, and derive the estimate of Rg from
	Hargreaves equation"
	"anInteger is the simulation day (so anInteger = 1 refers to 1 April"

"Hargreaves equation:
Rg = Ra * ah * sqrt(Tmax - Tmin) + bh
Rg		- Incoming daily global solar radiation [MJ m-2 d-1]
Ra		- Daily extra-terrestrial radiation [MJ m-2 d-1]
Tmax		- maximum temperature [oC]
Tmin		- minimum temperature [oC]
ah		- Empirical constant [°C-0.5]
bh		- Empirical constant [MJ m-2 d-1]
"

    | angot offset ah bh radX |
    offset := (Date newDay: 1 monthNumber: 4 year: year) dayOfYear. "91, in leapyear 92"
    angot := (angots at: anInteger + offset - 1).   "angots are in 10^7 J m-2 d-1"
    radX := angot * 10.0.                     "need to have it in MJ m-2 d-1 here!"
    "Use coefficients for Umea, Sweden, see www.supit.net appendix I"
    ah := 0.16.
    bh := 0.28 negated.
    rad := radX * ah * ((tempMax - tempMin) sqrt) + bh.
    "so with a delta T of 9, there is a reduction of factor 0.16 * 3 = 0.48, and a further subtraction of
0.28"
    "half of radiation is Photosynthetically Active Radiation (PAR)"
    par := rad * 0.48.
    ^par
```

Table S7 Calculation of day length (in hours), for a given day number, as coded in Smalltalk. Between brackets, EXCEL code (B column contains latitude; L column day number). Latitude in decimal degrees.

```
dayLengthForLat: latitude dayNr: aNumber
    | pi m dL lat dayN |
    pi := Float pi.
    lat := latitude.            "decimal degree"
    dayN := aNumber.            "the day number as required by the algorithm"

    "=1- TAN(B9*PI()/180) * TAN(23.439*COS(PI()/182.625*L9)*PI()/180)"
    m := 1.0 - ( ( lat * pi / 180.0) tan * ( 23.439* (( pi / 182.625 * dayN  ) cos ) * pi /180.0 ) tan ).

    "=24*ACOS(1-AL6)/PI()"
    dL := 24.0 / pi * ((1.0 - m) arcCos).
    ^dL
```

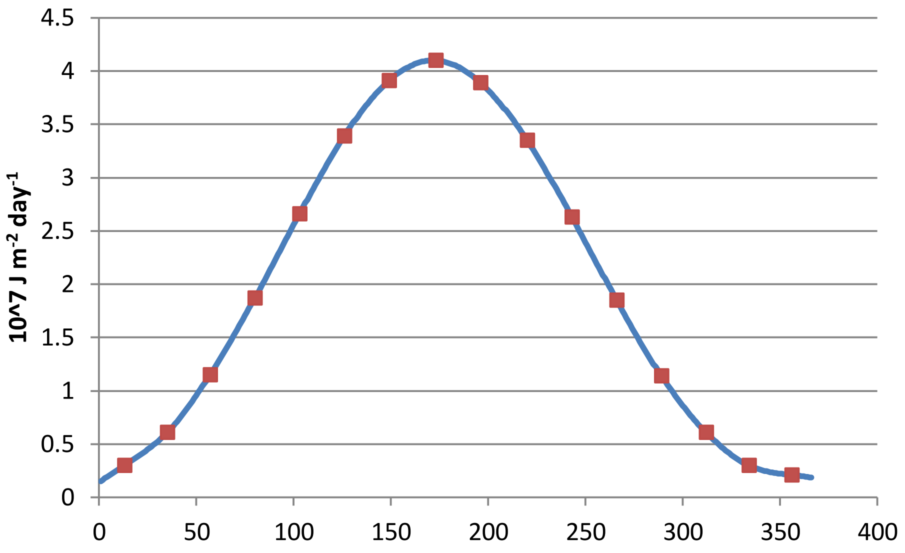

Fig. S5 Angot's values for 60° latitude.

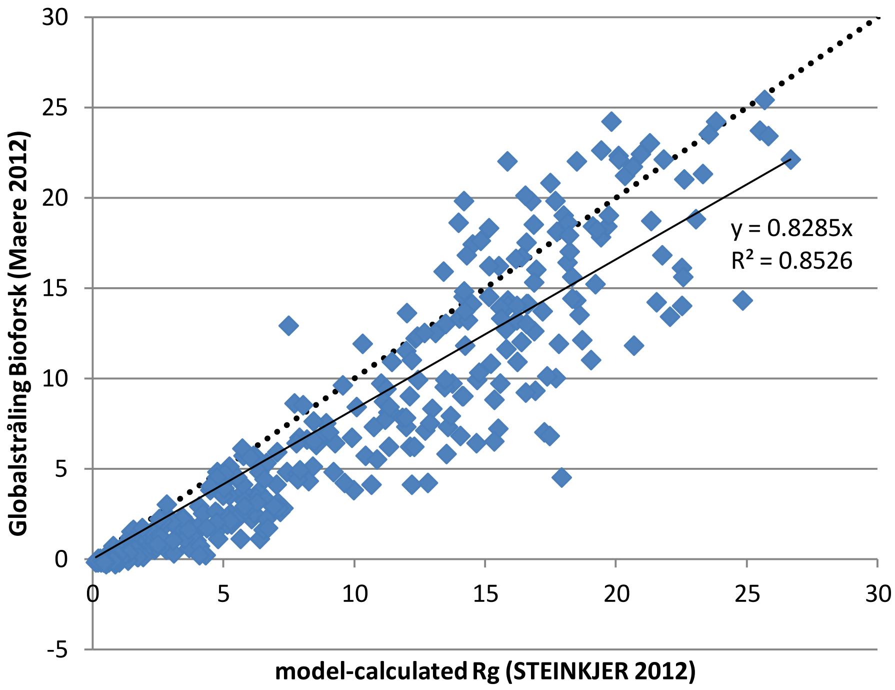

Fig. S6 Measured global radiation (MJ m^{-2} d^{-1}) at Bioforsk Mære station 2012 against model-calculated global radiation, from temperature data from Steinkjer weather station (71000). The model-generated values are somewhat underestimating the observed values (on average 83% of observed values).

1.8 Snow cover

From the data of snow disappearance on 4 weather stations (Table S8) in the area we derived an average last day with snow cover, at sea level (Table S9). By comparing these values with data from 3 other weather stations at higher altitude, an additional delay in disappearance of the snow cover was estimated to be approximately 1 day per 25 m interval (Bjerke, *personal comment*). Elevation values at the centre of each field were obtained from digital elevation model data at 50 m resolution (Norwegian Mapping Authority 2014).

Table S8 Weather stations in the area, used to estimate last day with snow from.

	Værnes	Mosvik-Trøahaugen	Verdal-Reppe	Utgård
Municipality	Stjørdal	Inderøy	Verdal	Steinkjer
Station no.	69100	71200	70150	70820
Alt.	12	39	81	50
UTM zone	33N	33N	33N	33N
X	297127	302771	336028	340298
Y	7043399	7082300	7077266	7114336

Table S9 Last day with snow cover and first snow-free day, for 4 weather stations, in 2009-2013. Værnes was considered as not representative for the whole area. Last column thus contains the values that were used in the model (day numbers 95, 97, 98, 104 and 107, respectively).

**Year**	Værnes	Mosvik - Trøahaugen	Verdal-Reppe	Utgård	Average date last day of snow	Range	Range w/o Værnes	Average date first snow-free day	Average date first snow-free day w/o Værnes
2009	29-3	3-4	1-4	9-4	2-4	11	8	3-4	5-4
2010	2-4	10-4	1-4	9-4	5-4	9	9	6-4	7-4
2011	3-4	6-4	6-4	11-4	6-4	8	5	7-4	8-4
2012	10-4	14-4	13-4	11-4	12-4	4	3	13-4	13-4
2013	12-4	14-4	14-4	20-4	15-4	8	6	16-	17-4

1.9 References

Bjerke, J. W., A. K. Bergjord, I. M. Tombre, and J. Madsen. 2013. Reduced dairy grassland yields in Central Norway after a single springtime grazing event by pink-footed geese. Grass and Forage Science.

Bonesmo, H. 1999. Modelling spring growth of timothy and meadow fescue by an expolinear growth equation. Acta Agriculturae Scandinavica - Section B Soil and Plant Science **49**:216-224.

Bonesmo, H., and G. Bélanger. 2002. Timothy Yield and Nutritive Value by the CATIMO Model Contrib. no. 714, Agric. and Agri-Food Can. Agron. J. **94**:337-345.

Chudzińska, M. E., F. M. van Beest, J. Madsen, and J. Nabe-Nielsen. 2015. Using habitat selection theories to predict the spatiotemporal distribution of migratory birds during stopover - a case study of pink-footed geese *Anser brachyrhynchus*. Oikos:n/a-n/a.

Mould, F. L. 1992. Use of a modified rising-plate meter to assess changes in sward height and structure. Norwegian Journal of Agricultural Sciences **6**:375-382.

Norwegian Institute of Bioeconomy Research. 2015. http://www.nibio.no/en.

Norwegian Mapping Authority. 2014. http://www.kartverket.no/en/Maps--Nautical-Charts/Gratis-kartdata/Open-and-Free-geospatial-data-from-Norway/

Simonsen, C. E. 2014. Goose/agricultural conflicts in Norway – building species distribution models: Objective tools in local management when allocating subsidies & evaluations on scaring cost effectiveness. Aarhus University, available at www.gint.no.

Statistics Norway. 2015. http://www.ssb.no/en/jord-skog-jakt-og-fiskeri/statistikker/stjord

2 RESULTS FOR RDM & SDM APPLIED TO NORD-TRØNDELAG

2.1 Resource Consumption & Accommodated Numbers

A

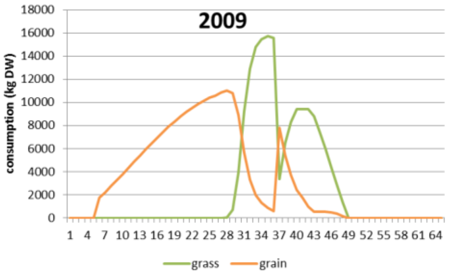

B

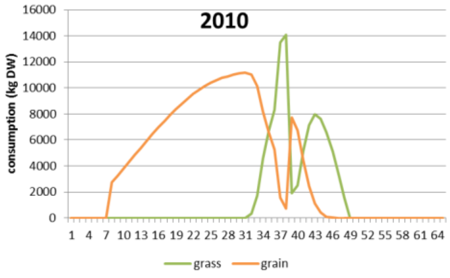

C

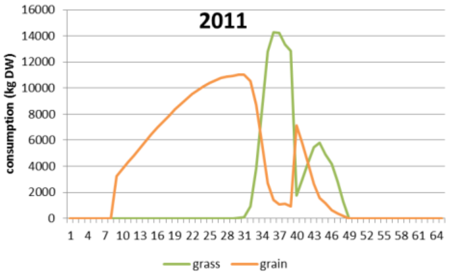

D

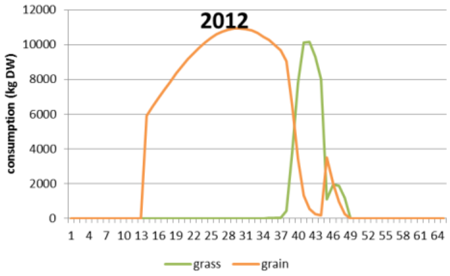

E

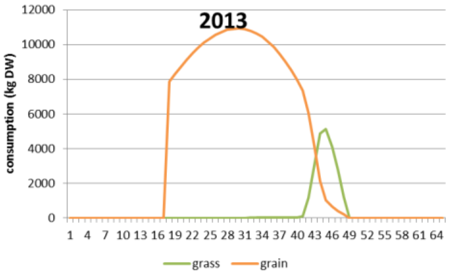

Fig. S7 A-E: model-predicted daily consumption (kg DW) of grass and grain by pink-footed geese in Nord-Trøndelag, mid Norway, for each of the 5 years. Average over 5 runs. Reference case: all fields available. Population sizes as estimated for the given year.

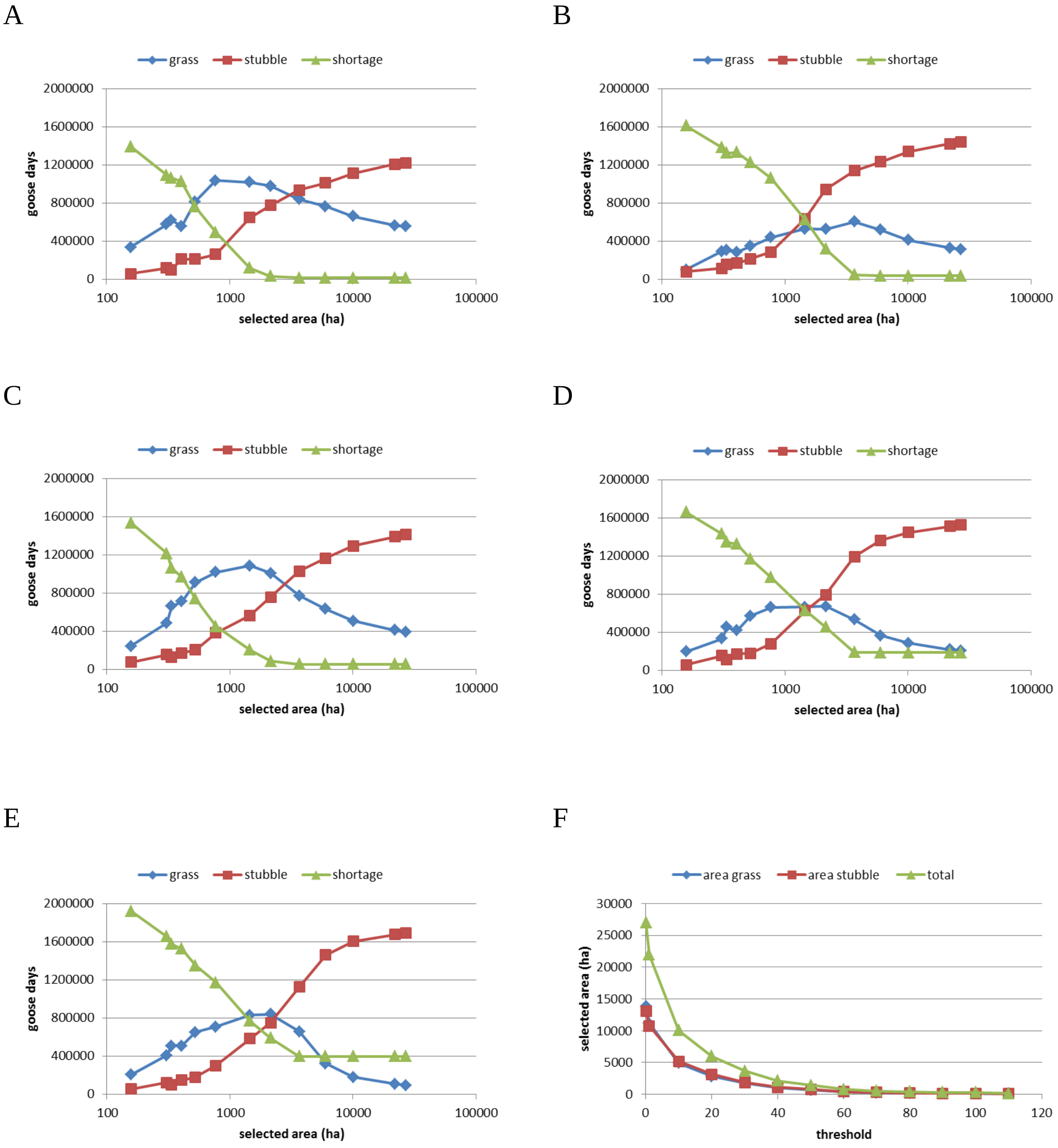

Fig. S8 Results of running the resource depletion model (RDM) on an increasing refuge area, when fields are added following the prioritization suggested by the species distribution model (SDM). Total numbers of goose days accommodated on grass and grain, and the shortage (unaccommodated goose days) for 2009 to 2013 (A-E). F: The relationship between selected refuge area distinguishing between grassland, cereal fields and total area, and the applied threshold value for dropping density. All values are averages over 5 runs.

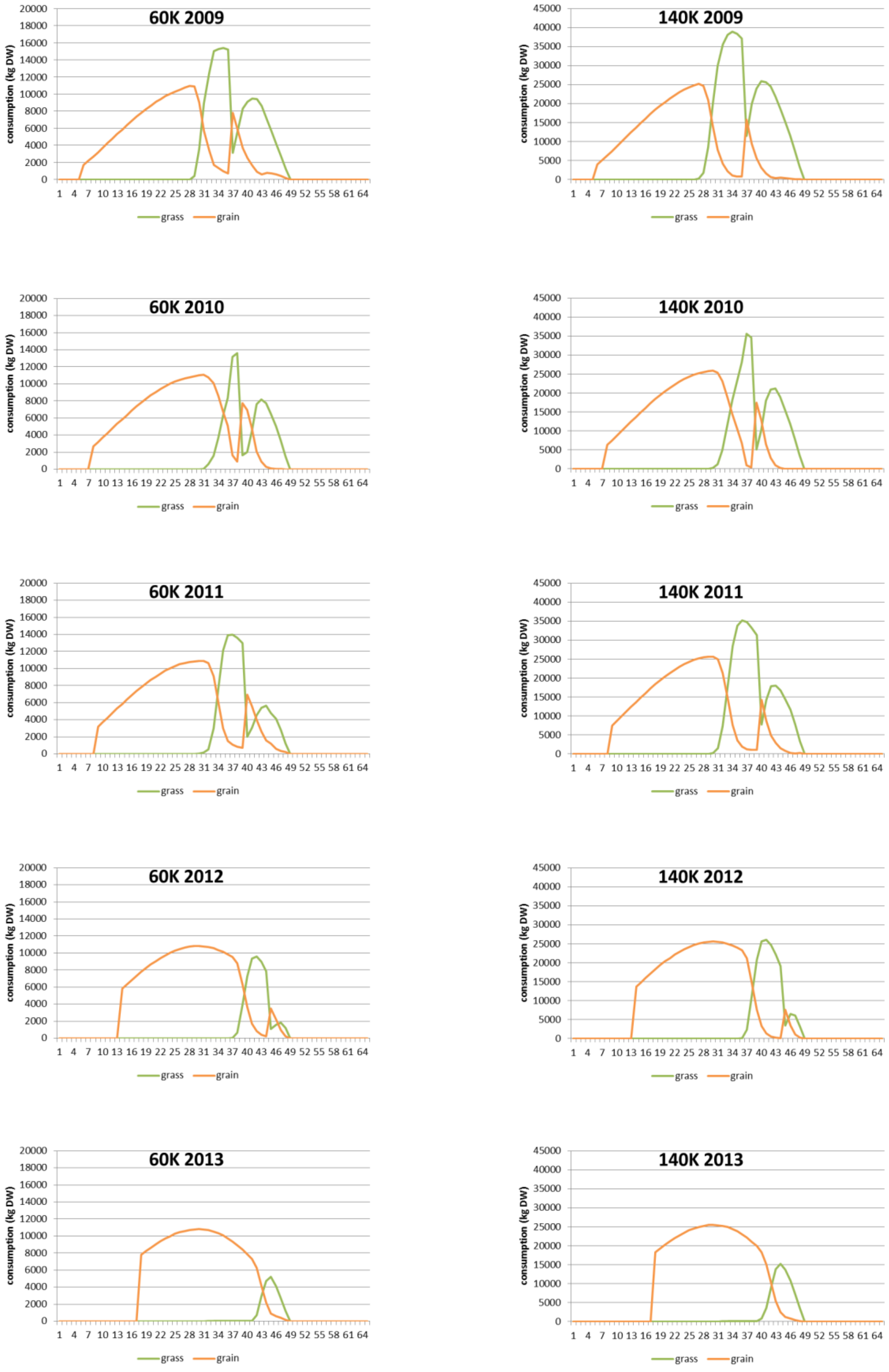

Fig. S9 Consumption of grass and grain resources, applying different weather sets ("2009" to "2013") and for 60K and 140K population sizes. For different population size, the patterns are identical but at a different scale. Reference case: all fields available.

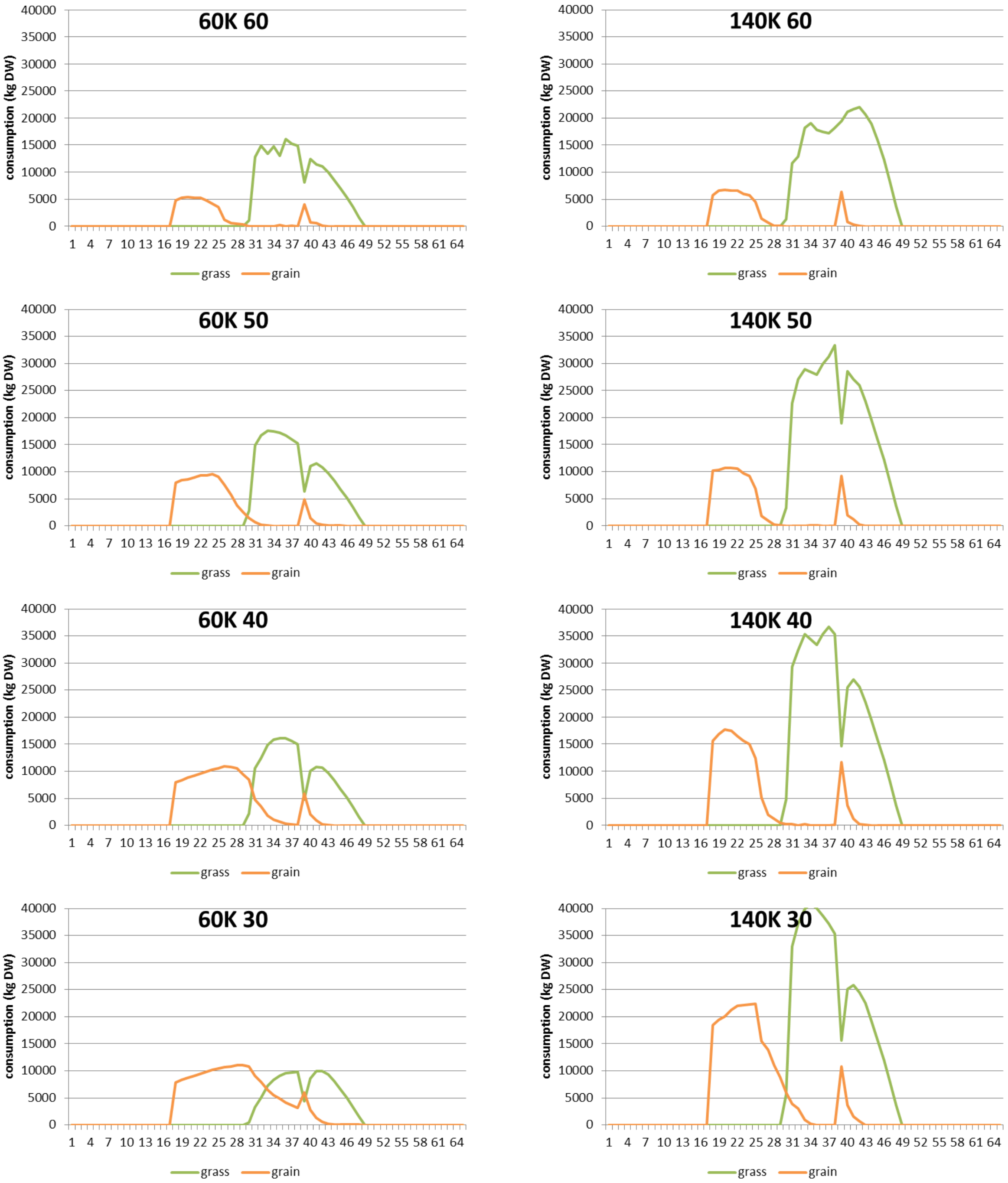

Fig. S10 Consumption of grass and grains during the staging period, for small (60K, left) and large (140K, right) maximum population size, and different refuge size. Refuges sizes of (top to bottom) 770 (60), 1457 (50), 2140 (40) and 3672 (30) ha. Between brackets the associated threshold in suitability values obtained from the species distribution model. The 2013 weather data set was used.

2.2 Comparison with Counts

Simulation results for 2010, 2012 and 2013 were compared to counts at the roosts, for corresponding dates. Results for 2011, cumulative over the whole period, were compared to the raw data underlying the SDM, dropping counts on a large number of fields.

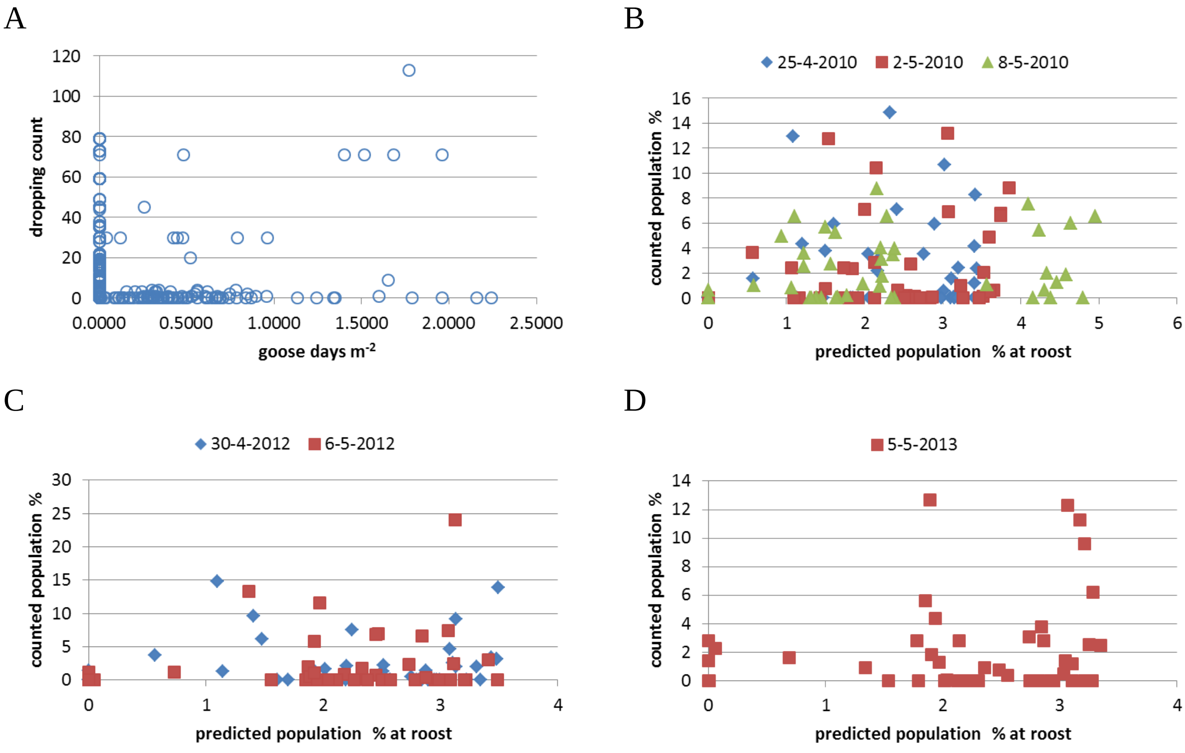

Fig. S11 A: Comparison of goose days m^{-2} observed in the simulations (averaged over 5 runs) with the dropping counts (2011). B-D: Comparison of predicted population at roosts (average of 5 runs) and counts at roosts, as percentage of the total population.

Roost counts

We calculated the root mean squared error (RMSE) for observed (Table S4) compared to simulated numbers at each roost:

(eq. 1)
$$RMSE = \sqrt{\frac{\sum(\hat{y}_i - y_i)^2}{n}}$$

Where y_i is the ith observation and $\hat{y}_i$ the corresponding predicted value (numbers at roost i). Results (Fig. S12) indicate that the fit does not improve when including fewer (and higher quality) fields in the set of available fields.

Dropping counts

Dropping counts were compared to total goose days per m^2 in the simulations, cumulative over the whole period. Due to different units, both were normalized on the maximum values (for counts and for goose days).

(eq. 2) $$RMSE\_normalized = \sqrt{\frac{\Sigma(\frac{\hat{y}_i}{\hat{y}_{max}} - \frac{y_i}{y_{max}})^2}{n}}$$

Results (Fig. S13) indicate that again the fit hardly increases when fewer (and higher quality) fields make up the set of available fields.

Known/Unknown land-use

As this may to some extent explain the limited fit between model and field data, we determined the fraction of the area with known land-use (cereal or grassland) and area of unknown land-use. Figure S14 showed that for almost all values of the threshold (and available area) more than half the area was of unknown land-use. Note that the values are different for each weather data set. This is caused by information on agricultural use that was available for more than one year, and differed between years. When this was the case the land use was selected for the year that matched the weather data set year.

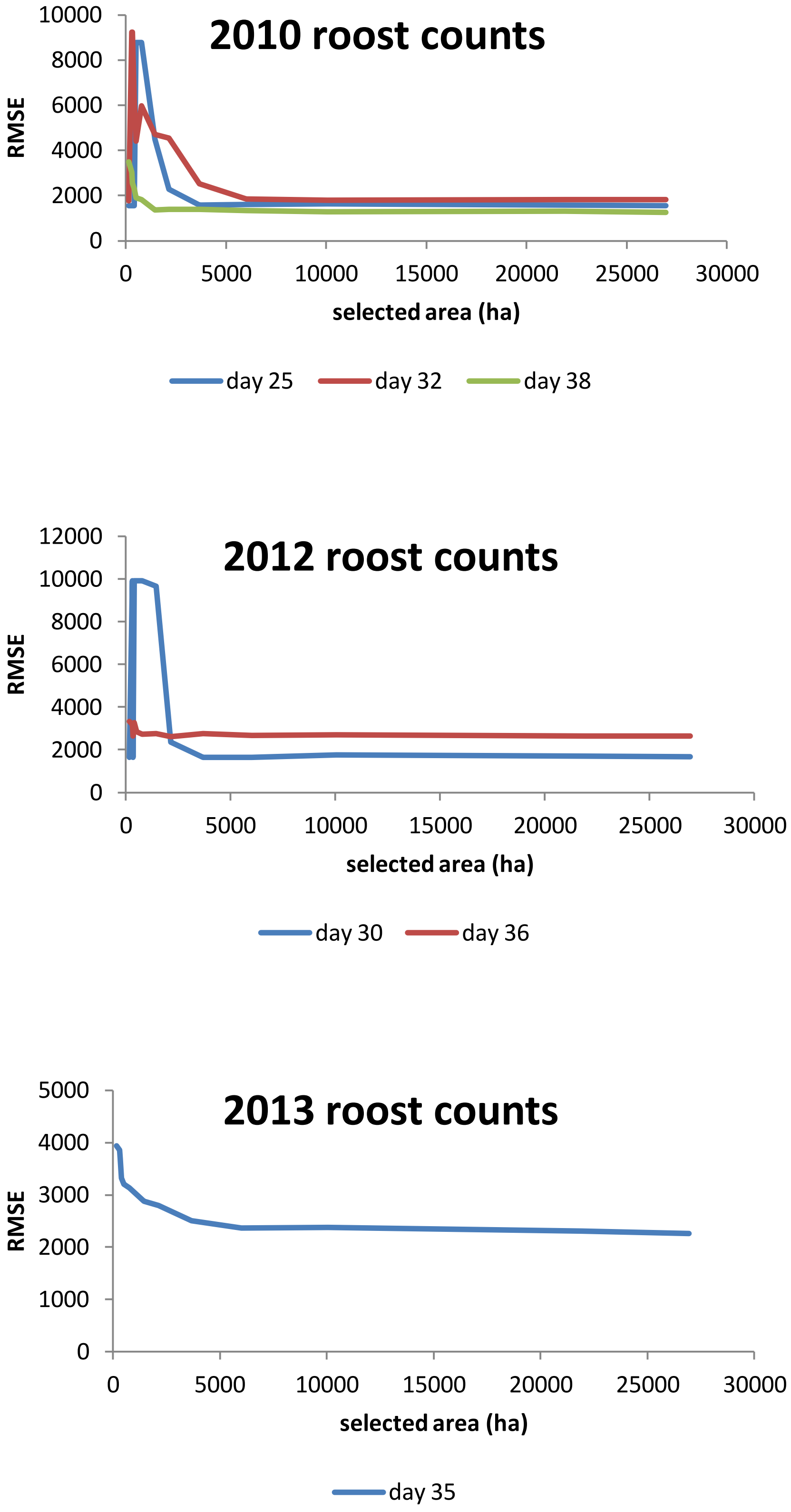

Fig. S12 The root mean squared error (RMSE) quantifying the fit between counts at roosts and model calculated values, over a range of refuge sizes. Refuge size was increased by adding fields in the order determined by the species distribution model.

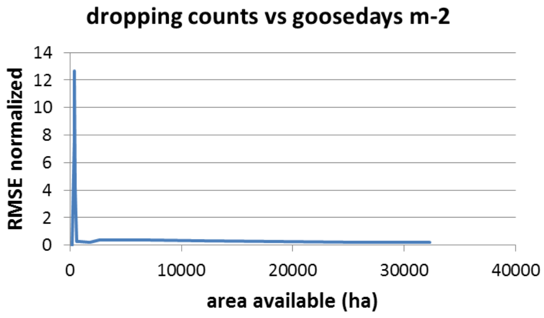

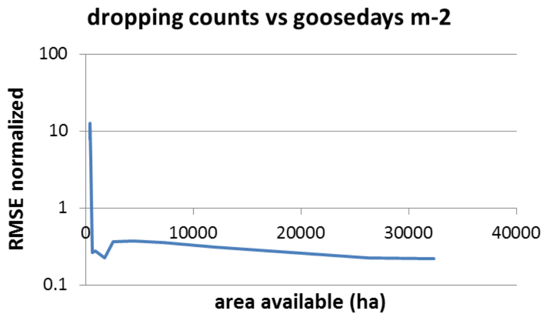

Fig. S13 The normalized RMSE (top) and the RMSE (bottom) over a range of refuge sizes (added in SDM-determined order), quantifying the fit between dropping counts and model calculated cumulated goose density in a limited number of fields.

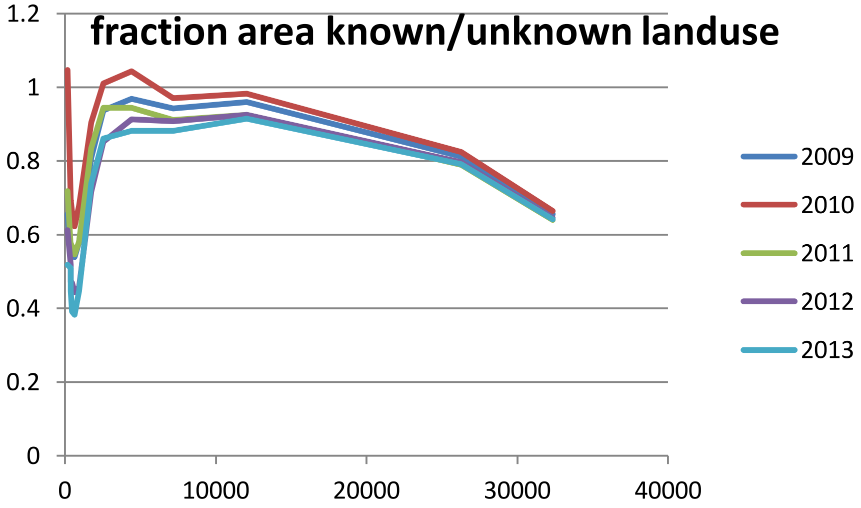

Fig. S14 Fraction of known/unknown land-use areas (ha/ha) for each of the years against refuge size.

2.3 Species Distribution Model

Table S10 Variables included (bold) and excluded in the final model

Variable	Explanation	Reason for exclusion in final model
**(gheight)**	**Grass sward height**	**Not available for entire region for model projections**
area	Size of field (m)	Low contribution to the model
**periarea**	**Perimeter/area ratio**	
water	Distance to open water (m)	Corr. with roost
**nonagri**	**Distance to roads and buildings**	
**roost**	**Distance to roost**	
**prcp4**	**Precipitation in April**	
tmax4	Max. temp. in April	Corr. with prcp4, tmin4 and dem
**tmin4**	**Min. temp. in April**	
dem	Elevation (m)	Corr. with prcp4, tmax4 and tmin4
slo	Slope (degrees)	Low contribution to the model
rad	Solar radiation	Low contribution to the model
**agri**	**Authority label on field (intensively grown crop or non-cultivated pasture)**	
**nb1000**	**% of available habitat in 1000m radius**	
nb500	% of available habitat in 500m radius	Corr. with nb1000, nb200 and nb100
nb200	% of available habitat in 200m radius	Corr. with nb1000, nb500 and nb100
nb100	% of available habitat in 100m radius	Corr. withnb1000, nb500 and nb200

Table S11 PQL mixed model results (lme4 model). See table S10 for variable explanations.

	Value	Std.Error	DF	t-value	p-value
**(Intercept)**	-3.076720	2.926741	203	-1.051244	0.2944
**prcp4**	0.068120	0.039548	203	1.722477	0.0865
**periarea**	-27.708361	8.866365	203	-3.125110	0.0020
**nonagri**	0.003354	0.001121	203	2.992324	0.0031
**roost**	-0.000669	0.000163	203	-4.095874	0.0001
**tmin4**	0.094762	0.080909	203	1.171215	0.2429
**nb1000**	0.027476	0.010378	203	2.647520	0.0087
**as.factor(agri)**	1.446459	1.009032	203	1.433512	0.1532

Table S12 Correlation between variables from the GLM model and PQL mixed model. See table S10 for variable explanations

	**prcp4**	**periarea**	**nonagri**	**roost**	**tmin4**	**nb1000**	**as.factor(agri)**
**prcp4**	-0.913						
**periarea**	-0.115	-0.066					
**nonagri**	0.056	-0.097	0.160				
**roost**	-0.065	0.052	0.108	-0.069			
**tmin4**	0.384	-0.434	0.011	-0.160	-0.070		
**nb1000**	-0.564	0.477	-0.031	-0.108	-0.220	0.061	
**as.factor(agri)**	-0.389	0.034	0.234	-0.006	0.057	-0.052	-0.012

3 SENSITIVITY ANALYSIS RDM APPLIED TO NORD-TRØNDELAG

We explored to what extent the capacity of the area to accommodate PFG and the resource consumption and potential yield loss caused by PFG depended on two selected coefficients, one that was considered relatively unknown (initial seed density on stubble fields) and another one that could be considered an important regulating parameter in the management: the fraction of stubble fields ploughed in spring. Default settings in the simulations presented so far were 408 seeds per m^2 and a 0.5 probability of spring ploughing.

Seed density varied in the analysis from 100 to 1000 seeds per m2; spring ploughing probability ranged from 0 (all fields ploughed in autumn) to 0.9. All combinations of these two coefficient values were tested, for two maximum population sizes (60K and 140K), and for all five weather patterns.

Results are shown in Figs. S15 and S16, for a maximum population of 60K, and in Figs. S17 and S18 for a maximum population of 140K.

A main conclusion is that the capacity changes hardly with changed seed density, and is affected only when the fraction of spring ploughing is very small. When both coefficients are small, however, the consumption of and the goose days accommodated on grass increases considerably (Figs. S15-S18).

Fig. S19 shows that the temporal pattern in resource consumption that leads to these cumulative values shown in figures S15-S18, may depend very much on the values of both coefficients, as these coefficients together determine the amount of grain resource that is available (Fig. S20).

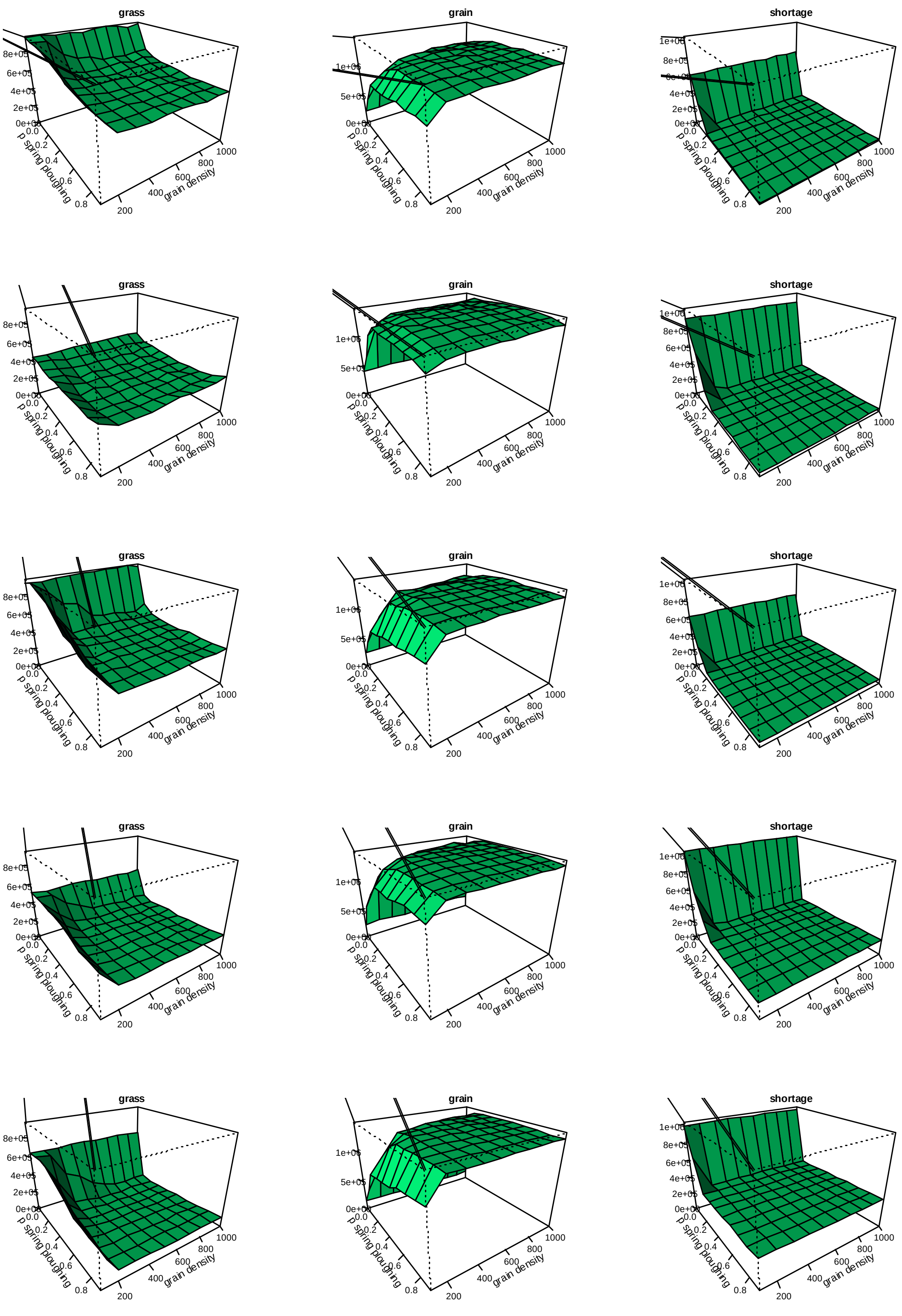

Fig. S15 Goose days accommodated on grass (left) and grain (middle), and the deficit (shortage, right) for different combinations of the probability of spring ploughing and the density of grains in cereal fields before ploughing. Maximum population size 60K. Top to bottom rows: 2009 to 2013 weather series.

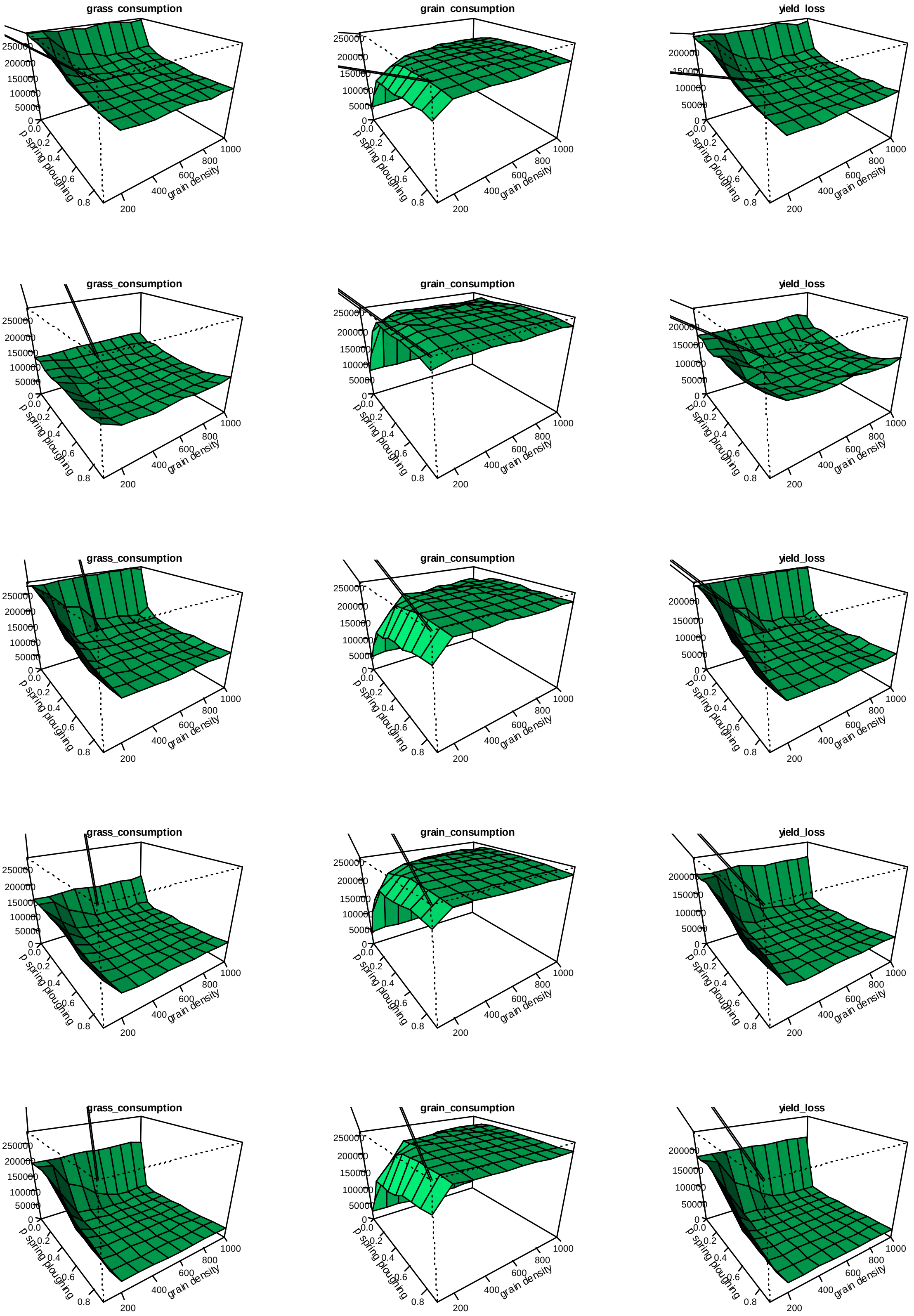

Fig. S16 Total consumption (kg) of grass (left) and grain (middle), and the yield loss (kg) at the end of the simulated period (shortage, right) for different combinations of the probability of spring ploughing and the density of grains in cereal fields before ploughing. Maximum population size 60K. Top to bottom rows: 2009 to 2013 weather series.

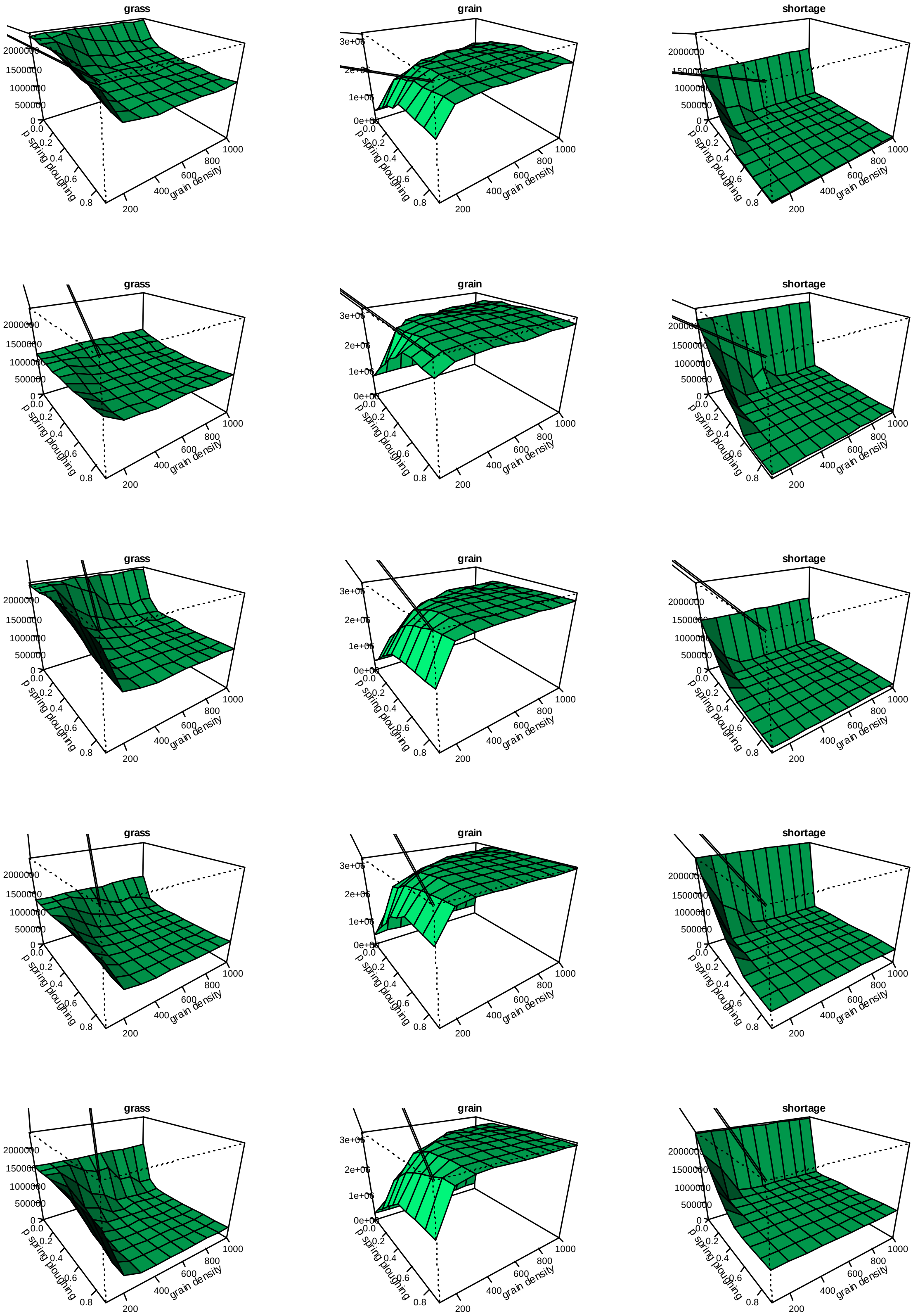

Fig. S17 As figure S15. Maximum population size 140K.

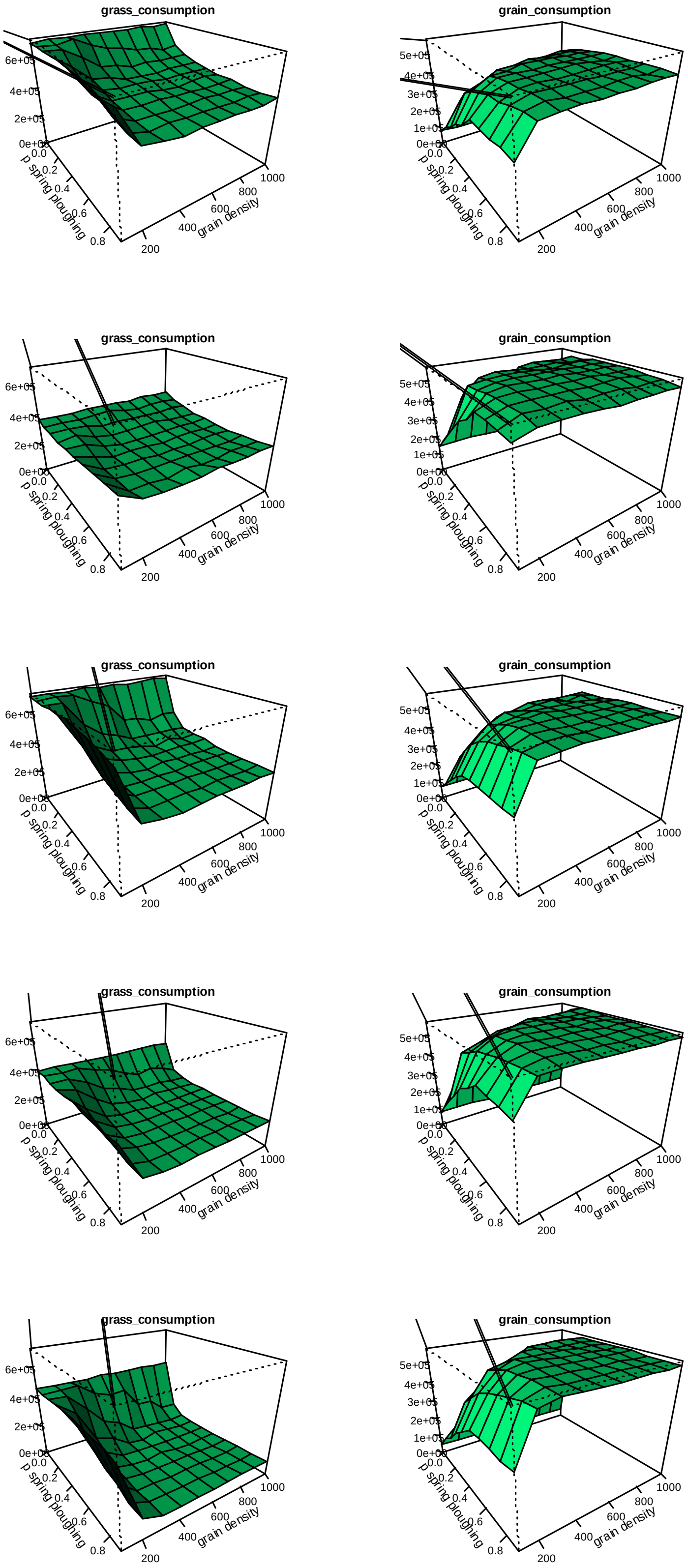

Fig. S18 As figure S16. Maximum population size 140K.

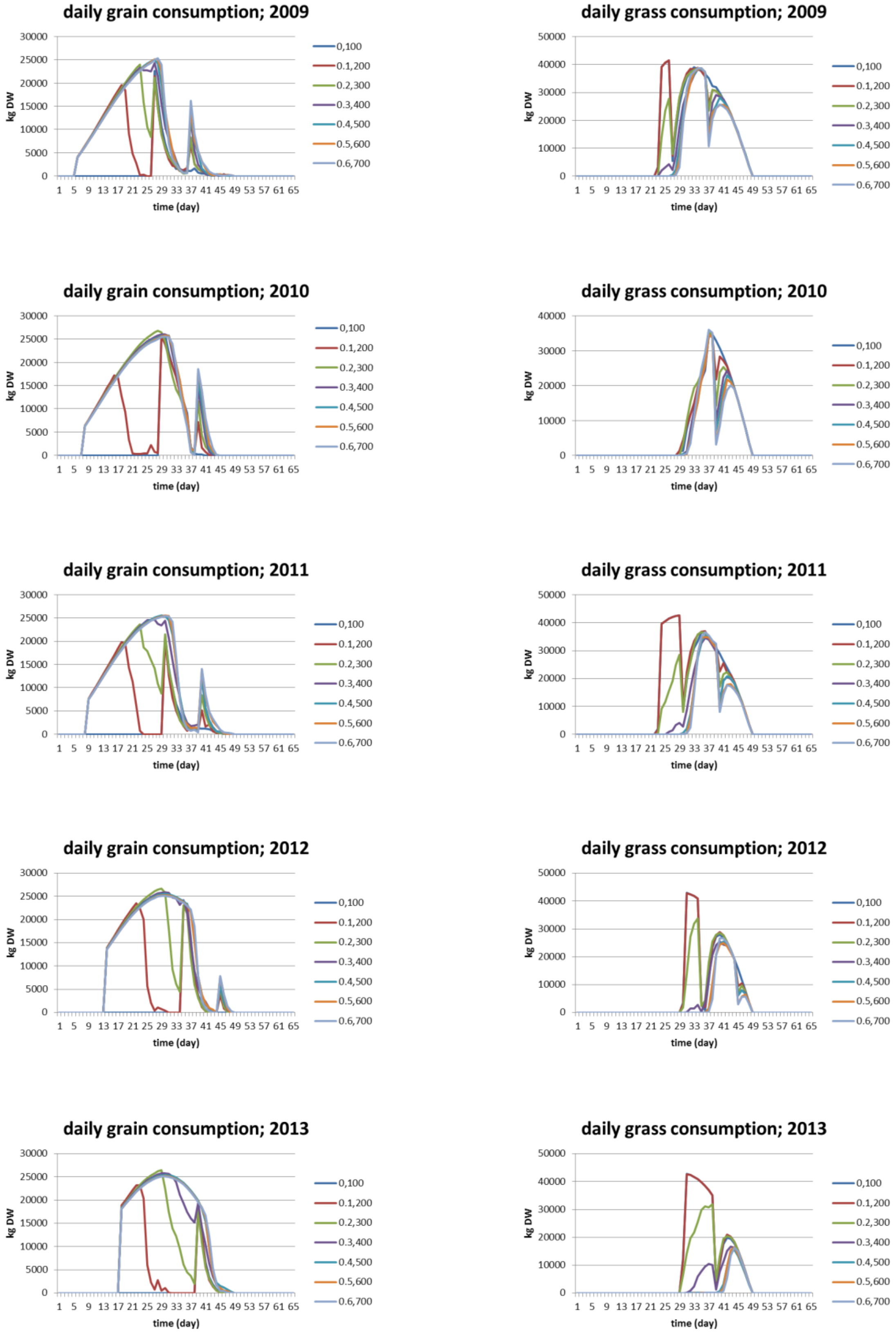

Fig. S19 For a selection of coefficient values (probability of spring-ploughing, initial density of seeds on stubble fields) following the steepest gradient (in Figs. S17 and S18) daily

consumption (kg DW) of grain (left) and grass (right), for each of the weather data sets 2009-2013 (top to bottom row). High maximum population size (140K).

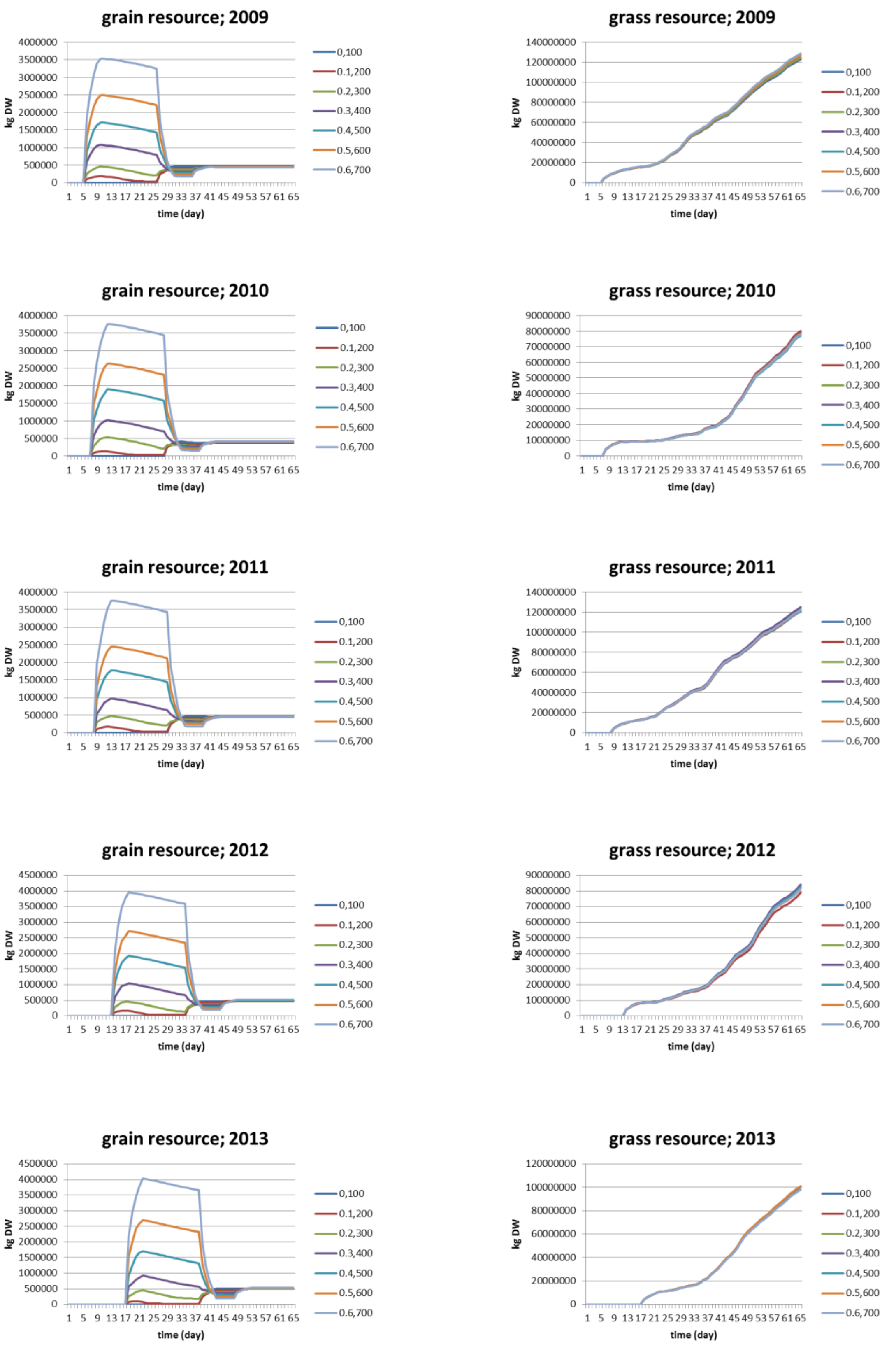

Fig. S20 Grain (left) and grass (right) resources (settings as in Fig. S19)
